# Release of Volatile Per- and Polyfluoroalkyl Substances from Plant Fiber-Based Food Packaging and Municipal Solid Waste to Gas under Simulated Landfill Conditions

**DOI:** 10.1021/acs.est.4c08544

**Published:** 2024-11-19

**Authors:** Yuemei Ye, Ivan A. Titaley, Mitchell L. Kim-Fu, Ansel R. Moll, Jennifer A. Field, Morton A. Barlaz

**Affiliations:** † Department of Civil, Construction, and Environmental Engineering, North Carolina State University, Campus Box 7908, Raleigh, North Carolina 27695-7908, United States; ‡ Department of Environmental and Molecular Toxicology, Oregon State University, 1007 Agriculture and Life Sciences Building, Corvallis, Oregon 97331, United States

**Keywords:** food packaging, municipal solid waste, landfill gas, volatile PFAS, leachate

## Abstract

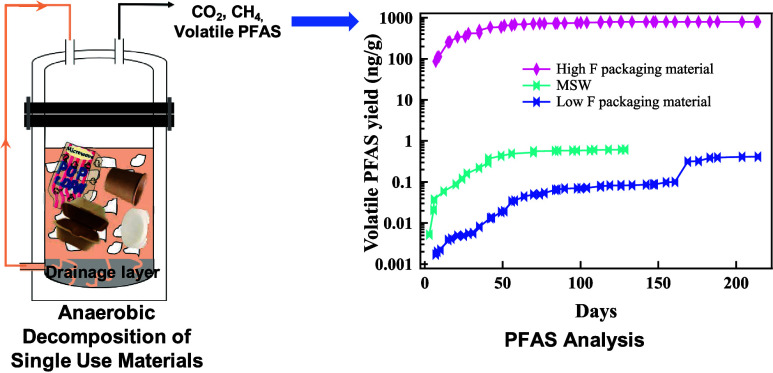

Per- and polyfluoroalkyl substances (PFAS) have been detected in plant fiber-based food packaging and most such packaging is disposed in landfills. The objective of this research was to evaluate the release of volatile PFAS to the gas-phase from PFAS-containing, single-use food packaging materials and from municipal solid waste (MSW) during anaerobic decomposition under simulated landfill conditions. After screening 46 materials for total *F* and 6:2 fluorotelomer alcohol (FTOH), packaging materials were classified as high or low *F*. High *F* materials included microwavable popcorn bags, natural plates, compostable bowls, biodegradable boxes, bagasse containers and eco-friendly plates, while the low *F* materials tested were paper plates, eco-friendly food trays and poly coated freezer paper. Summed PFAS release from the high *F* materials was 62–800 ng PFAS/g sample and 6:2 FTOH comprised 96.8–99.9% of the summed PFAS. The low *F* materials and MSW released 0.1–0.4 ng summed PFAS/g sample and 7:2-secondary (s) FTOH was the dominant volatile PFAS. PFAS were generally released early in the 123–285-day decomposition cycle, suggesting that some PFAS will be released prior to the installation of landfill gas collection systems. Nonetheless, PFAS have been reported in collected landfill gas, indicating that release occurs over many years.

## Abstract 2

This study demonstrates the release of volatile PFAS to the gas phase from PFAS-contaminated plant fiber-based food packaging under simulated landfill conditions.

## Introduction

The U.S. Environmental Protection Agency (USEPA) estimates that about 50% of municipal solid waste (MSW) is disposed in landfills.^1^ When MSW is disposed in a landfill, the waste biodegrades under anaerobic conditions, and CH_4_ and CO_2_ are generated. In addition to CH_4_ and CO_2_, other volatile constituents are released into what is referred to as landfill gas (LFG), i.e., the gas generated from waste decomposition.^2−4^ MSW includes many components that have been shown to contain per- and polyfluoroalkyl substances (PFAS), including for example, textiles,^5,6^ leather,^7^ paperboard,^8^ and food packaging.^8−12^

Landfill leachate contains a variety of ionogenic PFAS including PFSAs, perfluoroalkyl carboxylic acids (PFCAs), fluorotelomer carboxylic acids (FTCAs), and fluorotelomer sulfonic acids (FTSs), among others.^13−15^ In contrast to leachate, there is minimal information on volatile PFAS in LFG. To date, PFAS have been reported in LFG condensate^16,17^ and in the ambient air above landfills in Canada, China and Germany.^18−20^ PFAS detected include fluorotelomer alcohols (FTOHs), perfluoroalkyl sulfonamides, and fluoroalkyl sulfonamidoethanols, with 6:2 and 8:2 FTOH as the major components.^20^ Only recently was the presence of PFAS in LFG reported and the FTOHs dominated.^21,22^ The concentrations measured in LFG (830 000–4 900 000 pg/m^3^)^21,22^ are 2 orders of magnitude higher than those reported for ambient air above landfills.^18−20^

The presence of volatile PFAS in various types of consumer packaging that are routinely disposed in landfills has been reported.^10,11,23,24^ For example, FTOHs are associated with paper-based food packaging.^25^ Tian et al. reported 3000 ng 6:2 FTOH/g in “eco-friendly” food-contact material and 18 000 ng 6:2 FTOH/g in microwaveable popcorn bags purchased in the US,^19^ which are higher than concentrations in other consumer products.^8,9,12,25^ While the presence of PFAS on many items that are disposed in landfills has been documented, there are no data on volatile PFAS released to the gas under simulated landfill conditions. There is one report on the release of 6:2 FTOH from popcorn bags to the headspace of a water-filled container (55 °C), but this does not represent the physical, chemical or biological conditions of a landfill.^26^

The objective of this research was to evaluate the release of volatile PFAS to the gas-phase from a variety of PFAS-containing, single-use food packaging materials and from residential MSW during anaerobic decomposition under simulated landfill conditions. Initially, materials were screened to identify food packaging materials that contained volatile PFAS. Volatile PFAS release from selected materials was then measured under simulated landfill conditions in laboratory-scale reactors.

## Materials and Methods

### Material Collection and Characterization

**Figure 1:**
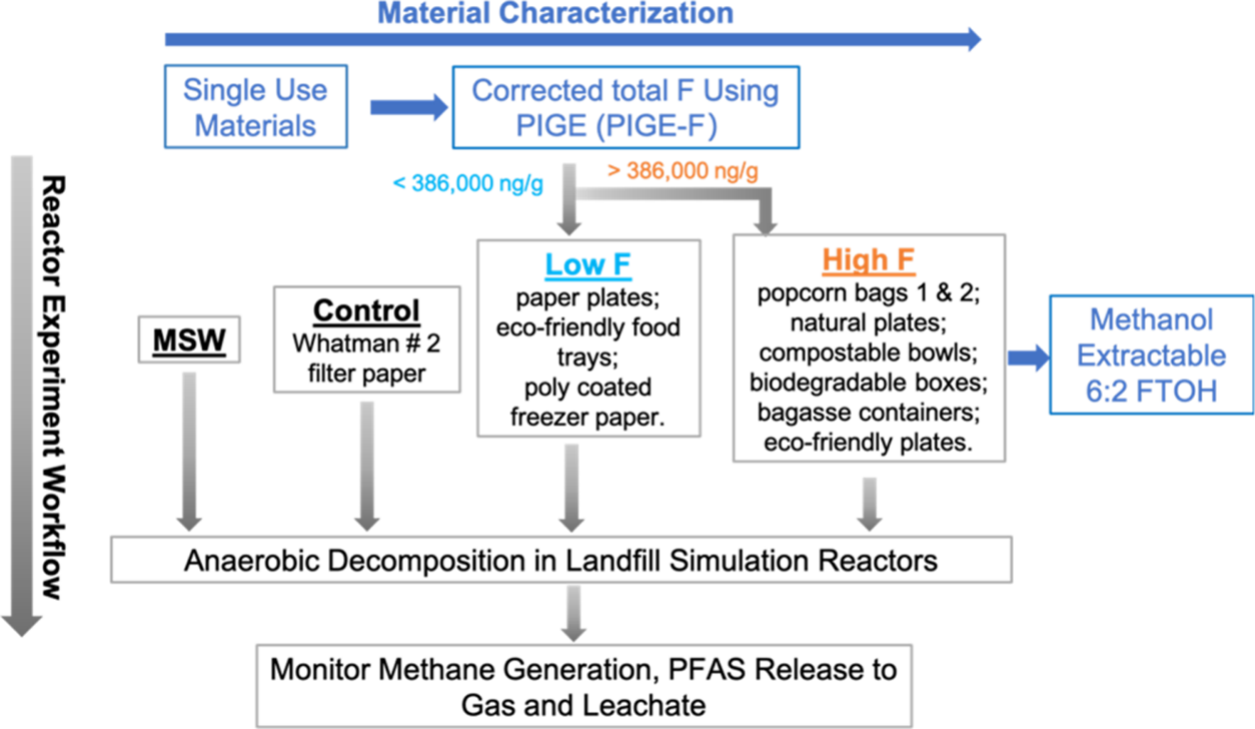
Overview of experimental program. All materials were screened by PIGE and those with >386 000 ng corrected *F*/g by PIGE (i.e., PIGE-*F*) were subject to a methanol extraction and 6:2 FTOH analysis. The high/low *F* criteria allowed for the division of potential test substrates and was otherwise arbitrary.

The overall experimental design is shown in Figure 1. Forty-six packaging materials, including paper plates, microwaveable popcorn bags, take-out food boxes, food wrappers, and various food containers were collected from local restaurants, online retailers, and grocery stores (Tables 1 and S1). While some materials were sold as a product (e.g., microwaveable popcorn), others were marketed based on their function (e.g., plate) or an environmental descriptor (e.g., eco-friendly). The popcorn was removed but the bags were not microwaved first which would have been appropriate to simulate a bag as disposed.

**Table 1:** Food Packaging Materials Selected for Study of PFAS Release in Landfill Simulation Reactors^a^

reactor group	no. of reactors	test material description^b^^,^^c^
high *F*	1	popcorn bags 1–6 brands of microwavable popcorn bags^c^
	1	popcorn bags 2–2 brands of microwavable popcorn bags^c^
	1	compostable bowls (1 brand)*
	1	biodegradable boxes (1 brand)*
	1	bagasse (sugar cane residue) containers (1 brand)
	3 (designated a, b, c)	natural plates (1 brand)*
	1	eco-friendly plates (sugar fiber is made with 100% nontoxic plant byproduct material) (1 brand)
low *F*^d^	2 (designated a, b)	paper plates + eco-friendly food trays (≥18% recycled wood fiber content) + poly coated freezer paper
control	2	Whatman #2 filter paper
MSW-May	2 (designated a, b)	fresh residential MSW collected May 2022
MSW-August	2 (designated a, b)	fresh residential MSW collected August 2022

a A list of all materials screened is given in Table S1.

b The material descriptions match the manner in which each material is marketed. Some materials are described by function (e.g., paper plate), while others are marketed as having a characteristic that is presumed desirable from an environmental perspective (e.g., eco-friendly food tray). A * by a test material means that the attribute describing the material has been certified by a testing agency. For example, compostable bowls were certified by the Biodegradable Products Institute.

c Materials were collected in 2021 with the exception of a second set of microwavable popcorn bags that was collected in 2022.

d The three low *F* materials were combined and tested in duplicate reactors.

The biochemical methane potential (BMP)^27^ of each selected material, as well as the cellulose, hemicellulose and lignin concentrations^28^ were measured to characterize the materials and ensure that each would biodegrade under anaerobic conditions (Table S2) (see Supporting Information (SI) for details).

Initially, samples were screened for total *F* by particle-induced γ ray emission (PIGE) spectroscopy after reducing their size to ∼2.5 cm × 2.5 cm.^5^ Samples that contained >386 000 ng corrected total *F*/g by PIGE (i.e., PIGE-*F*) were then screened for methanol-extractable volatile PFAS (Table S1).^29^ The popcorn bags used for PIGE and methanol extraction were cut from the edges of the bags, which were not in contact with the popcorn and oily residue. Prior to methanol extraction, samples were ground in a wiley mill to pass a 1 mm screen and spiked with internal standards (IS). The methanol extract was analyzed using an Agilent 7890B gas chromatograph (GC) and Agilent 5977A mass spectrometer (MS) for the analytes listed in Table S3.^29^

Fresh MSW was collected in spring (May) and summer (August) 2022 from the East Wake Transfer Station (Raleigh, NC). The MSW was shredded to ∼2 cm × 5 cm with a slow-speed, high torque shredder (ShredPax Corp., AZ-7H, Wood Dale, Illinois). Prior to shredding, the MSW was sorted to remove large pieces of metal, and large textiles were cut into smaller pieces so they could be processed through the shredder. The shredder was cleaned prior to use by shredding a ∼11L bucket of wood chips. About 25 kg of MSW was shredded and discarded, after which ∼250 kg was shredded twice, the pile was then mixed and a ∼6 kg subsample was transported to the laboratory for reactor loading. The MSW was stored at −4 °C prior to use.

### Reactor Construction, Loading, Operation, and Monitoring

Packaging materials (high and low *F*) selected for decomposition testing and Whatman #2 filter paper were shredded into ∼0.4 cm × 15 cm strips using a paper shredder prior to reactor loading. The shredder was cleaned with methanol, followed by shredding a few pieces of PFAS-free Whatman #2 filter paper before use and between samples.

Reactors were constructed using a two-piece glass system sealed with an ethylene propylene diene monomer gasket (Figure S1). Prior to loading, a pea gravel drainage layer covered with wire mesh was placed between the material and reactor outlet to protect the leachate outlet from clogging (Figure S1). All reactor parts were made from high-density polyethylene, polypropylene, poly(vinyl chloride), or polycarbonate, and all parts were rinsed three times with methanol before use. After filling with the test material, reactors were purged with N_2_ and a methanogenic inoculum was added to the system as described previously (see SI for details).^30^ Reactors were operated under conditions designed to maximize the rate and extent of anaerobic decomposition as described previously,^30^ which includes incubation at 37 °C and leachate recycle and neutralization. Leachate was recirculated once a week to accelerate decomposition. The leachate was neutralized weekly to ∼pH 6.8 as necessary to promote methanogenic activity.

The extent of material biodegradation was assessed by measuring gas generation and the corresponding methane concentration.^31^ Gas samples were collected from weekly to monthly, with higher frequencies when reactors were exhibiting relatively higher methane production. All gas data were corrected to standard temperature and pressure. To sample the gas for volatile PFAS, an internal standard (IS)-spiked universal sorbent tube (polymer, carbon black, and carbon black molecular sieve, Markes, Gold River, CA)^21^ was inserted between each reactor and its gas collection bag (Figure S2). Between 100 and 300 mL of generated gas was allowed to pass through the IS-spiked sorbent tube.^21^ The volume of gas that passed through the sorbent tube was collected in a 0.5 L gas bag located downstream of an acidified-water trap. The acidic water trap prevented the chemical exchange between the 0.5 L gas bag and the sampling tube and eliminated CO_2_ dissolution. Sampling tubes were stored at −4 °C prior to analysis.

For each reactor gas sampling event, two sampling tubes (sample controls) were uncapped during the sampling process and capped after sampling to check for volatile PFAS background. An additional sampling tube remained capped throughout the shipping process as a trip control. In the sample controls, there was an occasional (3 of 60 tubes) detection of 6:2 FTOH, 10:2 FTOH, N-MeFOSE, and N-EtFOSE. However, these compounds were never detected in the control reactors and therefore not considered to be a consistent system contaminant. There was never quantifiable PFAS in the trip controls.

Leachate samples (100 mL) were collected monthly and stored in 120 mL PFAS free polypropylene bottles at −20 °C. One bottle of DI water in a similar container was opened in the sampling area as a blank at each sampling. Only PFHxA and 10:2 FTS were detected with concentrations of 1.66–2.65 and 6.11 ng/L, respectively, and corrections were handled as described below.

For the high and low *F* materials, a subsample of the residual solids remaining after decomposition was subjected to methanol extraction and GC-MS analysis for volatile PFAS (see SI for details).^29^ The remaining solids were dried to constant weight at 75 °C for analysis of cellulose and hemicellulose to determine the extent of material decomposition. To evaluate PFAS loss to the reactor system at the end of the monitoring period, a methanol extract of the gasket from each reactor was analyzed for volatile PFAS. The gasket was extracted with 30 mL methanol in a falcon tube on a tube rotator at 65 rpm for 24 h. In addition, the inner wall of each reactor was rinsed with 50 mL methanol and analyzed for volatile PFAS by GC-MS.^29^

### Analytical Methods

Volatile PFAS in the gas samples were analyzed by thermal desorption-gas chromatography–mass spectrometry (TD-GC-MS) for 25 target and 17 suspect analytes (Tables S3 and S4).^21^ The TD-GC-MS system and its limit of detection (LOD) and quantification (LOQ) were previously described.^21^ Samples were brought to room temperature prior to analysis.

Leachate samples were subjected to solid phase extraction followed by analysis on an Agilent 1290 LC coupled to a 6495c Agilent QqQ using two separate methods as described in the SI. The first method consisted of a large panel of PFAS published previously,^33,34^ and the second method was an FTCA-only panel; 52 analytes are included in total for both methods (Table S5). Of the 52 compounds analyzed, 24 had average (*n* = 5) recoveries of 64–103% (observed concentration/expected concentration) (Table S5), therefore only these 24 compounds are presented.

The BMP of each material was measured to confirm its anaerobic biodegradability.^27^ In the BMP assay, materials that had been ground in a Wiley mill to pass a 1 mm screen were tested in 160 mL serum bottles containing 85 mL of growth medium and 15 mL of a methanogenic consortium.^27^ Cellulose, hemicellulose, and Klason lignin were also measured as described in the SI.^28^

### Verification of a PFAS-Free Reactor System

To confirm that the reactor system was PFAS-free, tests using both deionized water and N_2_ were conducted. Approximately 1 L of deionized water was added to a reactor and recirculated twice daily for 2 days, after which a water sample was analyzed for PFAS. No PFAS were detected using EPA 537 M for 21 ionic PFAS (Table S6).

To evaluate PFAS contamination attributable to the gas bag, 1 L of high purity N_2_ was injected into the bag, stored for 72 h and then sampled by collecting 300 mL through an IS-spiked TD tube. Three compounds (MeFOSE, EtFOSE, 10:2 FTAc) were detected < LOQ; however, they were never detected in the PFAS-free control (Whatman #2 filter paper) reactors. Therefore, the laboratory reactor system and the gas sampling process did not contribute background volatile PFAS.

Several PFAS were consistently detected in the leachate in the control reactors including PFBA (0.7–37.5 ng/L), PFPeA (0.19–21.25 ng/L), PFHxA (1.73–18.16 ng/L), PFOA (5.83–20.82 ng/L), and GenX (2–2.5 ng/L) (Figure S3). In addition, PFNA (14.35 ng/L), PFDoDA (9.45 ng/L), and PFTeDA (17.27 and 8.38 ng/L) were detected occasionally. With the exception of PFOA and 10:2 FTS, which were either not detected or detected at concentrations close to the background levels, the other PFAS were measured at concentrations from two to hundreds of times higher than the maximum concentrations detected in the control reactors. Concentrations were reported after subtracting the highest value measured in the controls. In the case of 10:2 FTS, which was detected in some sample blanks, it was never detected above its background in leachate.

### Data Analysis

The mass of volatile PFAS released to the gas phase was determined by multiplying the measured PFAS concentration by the measured gas volume. The volume of gas generated in each reactor was monitored more frequently (2–4 weeks) than the PFAS concentration. In cases where the gas volume was measured but the volatile PFAS concentrations were not, the volatile PFAS concentration was estimated by linear interpolation between measured PFAS concentrations. When a PFAS peak was < LOD, the concentration was assigned 0. In cases where the peak was between the LOD and LOQ, the estimated concentration was used in volatile PFAS mass release calculations.

The total released volatile and aqueous PFAS as *F* from each material in the reactor experiment was calculated by summing the *F* content from individual PFAS released in both the gas and aqueous phases. This summed total *F* was then divided by the corrected PIGE-*F* for the same sample before the decomposition reaction to evaluate the ratio of released *F* to the stored total *F* of each sample. Volatile *F* was calculated from the cumulative yield of individual PFAS in the gas phase at the end of the decomposition cycle, while aqueous *F* was determined from the highest release of individual PFAS measured in the leachate over the monitoring period.

## Results and Discussion

### Material Screening and Characterization

Based on the PIGE-*F* and 6:2 FTOH concentrations, the 46 materials were classified as high or low *F* (Table S1 and Figure S7). High *F* materials included 8 of 13 popcorn bags and 5 food packaging materials. Popcorn bags were tested in 2 sets (Popcorn Bags 1 and 2). Six high *F* popcorn bags collected in 2021 were combined as one mixed material (Popcorn Bags 1). As described below, it was necessary to restart the Popcorn Bags 1 reactor, and an additional 2 high *F* popcorn bags collected in 2022 were combined for another reactor test as there was not sufficient mass of the initial sample for retesting (Popcorn Bags 2).

Materials that did not exceed the high *F* thresholds were classified as low *F*. Interestingly, of the popcorn bags collected in 2022 (Popcorn Bags 2), 6:2 FTOH was only detected in two of six bags with concentrations of 8310 and 2470 ng/g, whereas six of seven of the popcorn bags sampled in 2021 (Popcorn Bags 1) were classified as high *F*. The reduction in the number of popcorn bags with detectable FTOH may be attributed to the ban enacted by 11 U.S. states on the use of PFAS in food packaging at the end of 2022.^32^ The presence of elevated volatile PFAS in five of the food packaging materials (natural plates, compostable bowls, biodegradable boxes, bagasse containers, eco-friendly plates) is consistent with recent reports showing that these materials contain high concentrations of total fluorine.^23,33^

The BMPs of the materials tested in reactors ranged from 129 to 342 mL CH_4_/dry g (Table S2), confirming their anaerobic biodegradability. The mix of six popcorn bags from 2021 (Popcorn Bags 1) and mix of two popcorn bags from 2022 (Popcorn Bags 2) exhibited similar BMPs (Table S2). All packaging materials contained greater than 70% cellulose plus hemicellulose in addition to lignin (Table S2).

### Decomposition of Tested Materials

**Figure 2:**
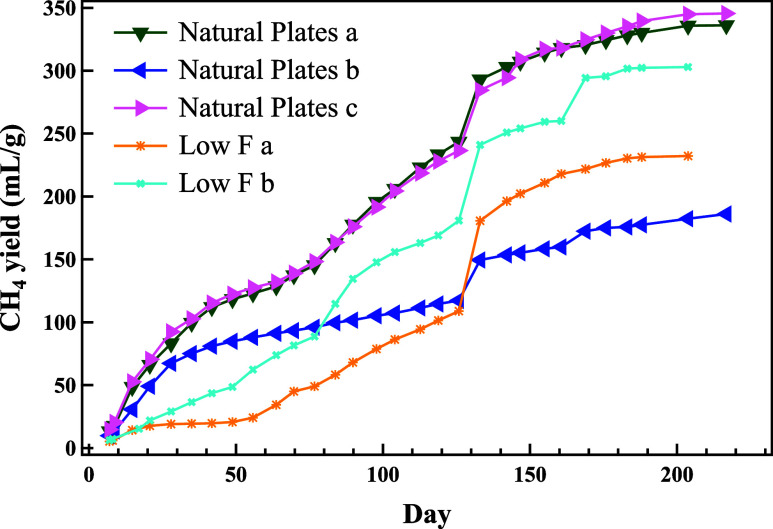
CH_4_ yields for natural plates and low *F* reactors.

The CH_4_ yield for selected materials is presented in Figure 2. The yields for the remaining materials as well as CH_4_ generation rates and reactor pHs for all tested materials are presented in Figures S4–S6. The test materials demonstrated high extents of cellulose (>52%) and hemicellulose (>73%) loss (Table S7). Biodegradation in Popcorn Bags 1 became inhibited and the reactor was discontinued after 27 days (Figures S5C and S6C). A second popcorn bag test was then initiated and exhibited high conversion to CH_4_ (Figures S5C and S6C). The BMPs of the first and second popcorn bags samples were similar (Table S2) and the reason for the inhibition is unclear.

### Release of Volatile PFAS from the High *F* Materials

**Figure 3:**
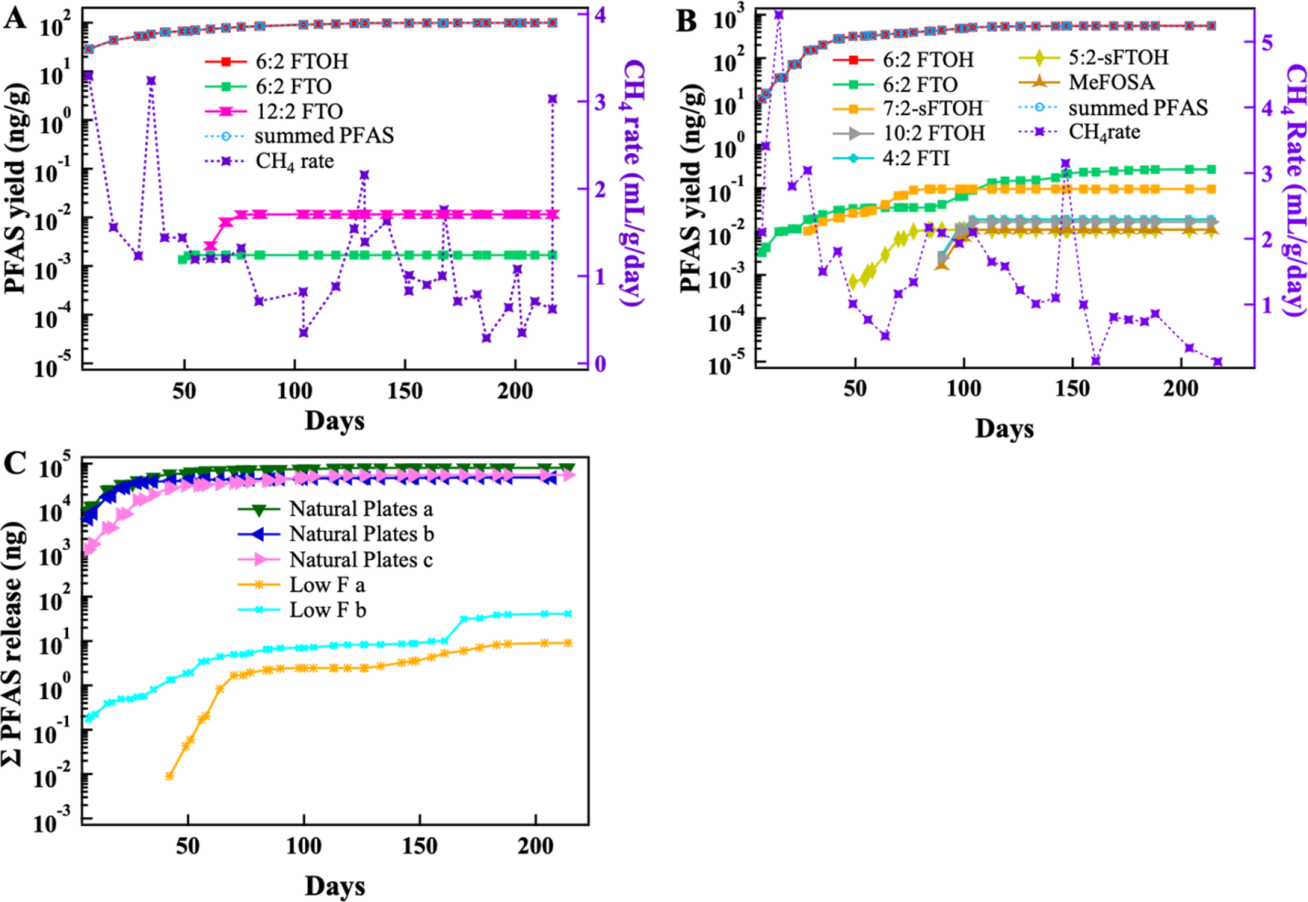
Time-dependent release of volatile PFAS to the gas phase under simulated landfill conditions in (A) Popcorn Bags 2, (B) Natural Plates c, and (C) summed volatile PFAS of all 3 Natural Plates reactors and Low *F* a and b reactors. The summed PFAS and 6:2 FTOH lines overlap in (A, B).

PFAS release from materials categorized as high *F* is summarized in Table 2, Figures 3 and S8–S10 and the data are presented in a spreadsheet attached to the SI. 6:2 FTOH was the dominant volatile PFAS released from all high *F* materials, comprising 96.8–99.9% of the summed PFAS. The yields of 6:2 FTOH varied from 62 to 800 ng/g (Table 2). In all high *F* reactors (Figures S8–S10), 6:2 FTOH was detected in the gas phase within 10-days of reactor initiation.

**Table 2:** Cumulative Volatile PFAS Yield in High *F* Reactors (ng/g Material)^a^

	reactors								
							Natural Plates		
PFAS (ng/g)	Popcorn Bags 1	Popcorn Bags 2	Compostable Bowls	Biodegradable Boxes	Bagasse Containers	Eco-friendly Plates	*a*	*b*	*c*
4:2 FTOH	0.0021	<LOQ	0.034	0.23	<LOQ	<LOQ	<LOQ	<LOQ	<LOQ
6:2 FTOH	14.6	99.7	341	474	121	61.7	798	487	555
8:2 FTOH	0.0062	<LOQ	0.033	0.38	<LOQ	<LOQ	<LOQ	<LOQ	<LOQ
10:2 FTOH	0.025	<LOQ	0.18	0.41	<LOQ	<LOQ	<LOQ	<LOQ	0.017
12:2 FTOH	<LOQ	<LOQ	<LOQ	1.04	<LOQ	<LOQ	<LOQ	<LOQ	<LOQ
MeFOSA	0.0045	<LOQ	0.009	0.039	0.0064	<LOQ	<LOQ	<LOQ	0.011
EtFOSA	0.0079	<LOQ	0.0051	0.0066	0.0081	<LOQ	<LOQ	<LOQ	<LOQ
N-EtFOSE	<LOD	<LOD	<LOD	<LOD	<LOD	<LOQ	<LOQ	<LOQ	<LOQ
6:2 FTAc	0.00044	<LOQ	0.00023	0.025	0.002	<LOQ	<LOQ	<LOQ	<LOQ
8:2 FTAc	0.038	<LOD	0.00098	0.14	<LOQ	<LOD	<LOQ	<LOD	<LOQ
10:2 FTAc	0.0068	<LOD	<LOQ	0.044	<LOQ	<LOD	<LOQ	<LOQ	<LOD
6:2 FTMAc	<LOQ	<LOQ	0.019	0.035	0.011	<LOQ	<LOQ	<LOQ	<LOD
8:2 FTMAc	0.01	<LOQ	0.00098	0.02	0.0017	<LOQ	0.0066	<LOQ	<LOQ
6:2 FTO	0.84	0.0017	1.46	2.6	3.46	0.24	0.23	0.13	0.27
8:2 FTO	0.0055	<LOQ	<LOQ	0.15	0.013	0.0045	0.0015	<LOQ	<LOQ
10:2 FTO	0.0016	<LOQ	<LOQ	0.1	0.0036	0.0022	0.0018	<LOQ	<LOQ
12:2 FTO	0.003	0.011	<LOQ	0.12	<LOQ	0.0030	<LOQ	<LOQ	<LOQ
PFHxI	4.7	<LOD	<LOQ	3.9	<LOQ	0.013	<LOQ	<LOQ	<LOQ
PFOI	0.081	<LOD	<LOQ	0.14	<LOQ	0.039	<LOQ	<LOQ	<LOQ
PFDI	<LOD	<LOD	<LOD	<LOD	<LOD	<LOD	<LOQ	<LOQ	<LOD
4:2 FTI	0.045	<LOD	0.0033	0.06	0.0063	0.0027	<LOQ	<LOQ	0.019
6:2 FTI	0.008	<LOQ	0.01	0.14	0.0079	0.004	<LOQ	<LOQ	<LOQ
14:2 FTOH	<LOQ	<LOD	<LOD	0.018	<LOD	<LOD	<LOD	<LOD	<LOD
5:2-sFTOH	<LOQ	<LOD	0.09	0.14	<LOQ	<LOQ	0.15	<LOQ	0.011
7:2-sFTOH	0.083	<LOQ	0.0061	0.16	<LOQ	0.021	0.7	0.086	0.096
∑PFAS	20.4	99.7	343	484	125	62	799	487	556

a If a compound is LOD in every case, then it was not included.

Prior work on aerobic biotransformation of landfill-leachate sediment reported the release of FTOH to the gas phase.^34^ Goukeh et al. also indicated that degradation of side-chain fluorinated polymers in consumer products release FTOHs to the gas-phase.^26^ Hydrolysis and biodegradation of side-chain fluorinated polymers, known to be used on food packaging,^35^ were shown to release clathrate-bound FTOHs as the degradation products.^36,37^ Therefore, the observed 6:2 FTOH in the gas-phase was likely due to decomposition processes that released FTOHs by either biodegradation or hydrolysis.^36,37^ However, plots of the natural log of 6:2 FTOH molar concentration with time did not yield a linear relation (data not shown), thus the data collected did not conform to a first-order reaction, as reported by others for side-chain fluoropolymer abiotic hydrolysis in water.^36^

The summed PFAS released from the triplicate reactors containing natural plates was consistent over time (Figures 3B and S9). In Natural Plates a (Figure S9A) and c (Figure 3B), transformation products of FTOHs, namely 5:2- and 7:2-sFTOHs, were detected in the gas, while only 7:2-sFTOH was detected in reactor b (Figure S9B). Perfluorohexyl iodide (PFHxI) is a nonpolymer fluorotelomer family member^38^ and was detected in Popcorn Bags 1 (Figure S8) and Biodegradable Boxes (Figure S10B) reactors, with yields that are ∼2 orders of magnitude higher than from Eco-friendly Plates (Figure S10D) reactor. Poly(fluorinated iodides) (PFIs) are intermediates and products of the telomerization process for FTOH synthesis.^39^ Both 4:2 and 6:2 FTI, which are reported to be precursors to FTOHs,^40^ were detected in all of the high *F* reactors except Popcorn Bags 2, and 2 of the 3 Natural Plates reactors (Table 2).

The Popcorn Bags 1 reactor was terminated after 27 day as methane generation became inhibited (Figure S5C) and the objective was to measure PFAS release throughout the decomposition cycle. Nonetheless, the rapid release of PFAS from the Popcorn Bags 1 reactor provides interesting information. Specifically, 19 volatile PFAS were released to the gas, with individual PFAS yields ranging from 0.0016 to 14.6 ng/g (Table 2 and Figure S8). Among volatile PFAS, 6:2 FTOH was the dominant (∼72%) volatile PFAS over the 27 day monitoring period. The other volatile PFAS were released from Popcorn Bag 1 reactor at three time points. On day 5, 6:2, and 8:2 FT-olefins (FTOs), 4:2 FT-iodide (FTI), and EtFOSA were released, but their yields were 2–4 orders of magnitude lower than that of 6:2 FTOH, which was released on day 12. Similar to FTIs, FTOs are also reported to be precursors to FTOHs,^40^ therefore, they could be synthesis residuals contained in the samples that were subsequently released to the gas. Nine additional volatile PFAS were first detected in a day 12 sample (Figure S8). The remaining three volatile PFAS, 8:2 and 10:2 FTAc, and 8:2 FT-methyl acrylate (FTMAc), were first observed in the day 18 sample. The FTAcs and FTMAcs have been reported to be present in paper-based food packaging.^23^

A second popcorn bags reactor was initiated with a different set of bags as there was not sufficient material remaining from the original popcorn bag sample. In contrast to Popcorn Bags 1 reactor, only three PFAS were detected during decomposition of Popcorn Bags 2 reactor: 6:2 FTOH was detected early, while 6:2 and 12:2 FTO were detected at ∼ day 50 (Figure 3A, Table 2). The 6:2 FTOH was again the dominant PFAS, accounting for 99.9% of the volatile PFAS released during the 203-day monitoring period. Most of the 6:2 FTOH released from both Popcorn Bags 1 and 2 was measured in the initial stage of decomposition, after which there was a gradual increase throughout the 27- or 203-day monitoring period (Table S8).

For Popcorn Bags 1 (collected in 2021) materials, some oily residue remained on the inside surface of the bags while the residue was removed from Popcorn Bags 2 materials using PFAS-free Kimwipes. Whether this difference explains the rapid release of 19 compounds from Popcorn Bags 1 cannot be determined from the available data and more investigation is required as there are other confounding factors. First, we did not control for storage time, which was shown to impact the presence of volatile PFAS in packaging materials.^23^ Second, 11 U.S. states banned the use of PFAS in food packaging at the end of 2022; thus, the bag composition may have changed.^32^

The concentrations of 6:2 FTOH measured in the gas samples from high *F* reactors ranged from < LOD to 5630 ng/L (Table S9), which is up to 3 orders-of-magnitude higher than the 6:2 FTOH concentration reported in LFG (0.83–4.9 ng/L).^19^ Higher concentrations in reactors containing high PFAS materials relative to LFG would be expected since there are many non-PFAS containing materials contributing to LFG from landfills, thus, diluting FTOH concentrations.

In general, PFAS release occurred early in the monitoring period and release plateaued prior to methane generation with the exception of the natural plates where methane generation peaked before 6:2 FTOH release (Figures 3, S9–S11). There were a few cases in which a spike in methane generation corresponded to a spike in the release of a volatile PFAS. For example, 4:2 and 10:2 FTOH and CH_4_ increased concurrently in the Compostable Bowls reactor at about day 180, 6:2 FTOH and CH_4_ in the biodegradable boxes reactor at about day 90, 6:2 FTI and CH_4_ in the bagasse containers reactor at about day 75, and 6:2 and 8:2 FTO with CH_4_ in the Eco-friendly Plates reactor early in the monitoring period (Figure S10). While the general trend was for PFAS release to occur early and to then plateau, there were exceptions such as the delayed spike in 4:2 FTI, 7:2-sFTOH, and 8:2 FTO in the reactor containing eco-friendly plates (Figure S10D). The increasing CH_4_ generation early in the decomposition period likely purged readily releasable PFAS from the test materials (Figure S10).

## Release of Volatile PFAS from the Low *F* Materials

PFAS release from the materials categorized as low *F* is summarized in Table S10 and Figure S11 and in a spreadsheet attached to the SI. The low *F* materials released ∼4 orders of magnitude less summed PFAS than the high *F* materials and fewer compounds were detected. Notably, 6:2 FTOH, which was the dominant PFAS released from the high *F* materials, was < LOD and 0.0066 ng/g in the 2 low *F* reactors. The dominant volatile PFAS released from the low *F* materials was 7:2-sFTOH which is reported to be the most abundant transformation product of 8:2 FTOH under aerobic conditions.^34^ The 8:2 FTOH was detected in the gas from Low *F* reactor b on day 43 and 7:2-sFTOH was first detected on days 74 and 51 in Low *F* reactors a and b, respectively. However, the 8:2 FTOH yield is well below that of the 7:2-sFTOH. Trace amounts of 10:2 FTOH (0.019 and 0.004 ng/g) were released from Low *F* reactors a and b on days 148 and 43, respectively (Figure S11A,B).

## PFAS Release from Municipal Solid Waste

**Figure 4:**
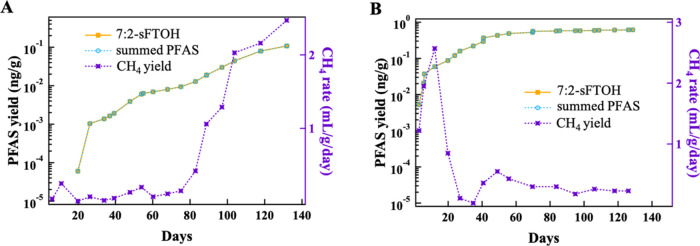
Time-dependent release of PFAS in MSW collected in May and August to the gas phase in reactors (A). MSW-May b, and (B). MSW-August a. The summed PFAS and 7:2-sFTOH lines overlap.

Duplicate reactors containing MSW sampled in both May and August were monitored and methane generation rate data are presented in Figures 4 and S11C,D. One of the reactors from the May sample (MSW-May a) was inhibited and monitoring was discontinued after 108 days, while methane generation in MSW-May b and the two reactors with MSW from August went through a typical decomposition cycle. PFAS release showed similarities to the low *F* materials, displaying low volatile PFAS release (Table S10). Notably, the dominant volatile PFAS released from the MSW reactors was 7:2-sFTOH, while 8:2 FTOH was < LOQ. It is possible that the detected FTOHs were from the degradation of the phosphoric diester acids—which have been reported in food contact materials.^23,33,41−44^

Given the presence of food packaging in MSW,^45^ and the observation that 6:2 FTOH dominates landfill gas (LFG),^21^ 6:2 FTOH was expected to be released from the MSW samples, but was instead < LOQ in all MSW samples (Table S10). The 10:2 FTOH was only detected from one of the MSW reactors while the potential product, 7:2-sFTOH, was detected in all MSW reactors (Table S10) The summed PFAS in the MSW reactors was 0.08–0.15 ng/g, which is ∼2 orders of magnitude less than what was measured for the high *F* materials but comparable to PFAS release from the low *F* materials. Because MSW has many anaerobically degradable components that do not contain PFAS, lower PFAS release relative to the high *F* materials was expected. Interestingly, 14 000 and 8000 ng 6:2 FTOH/g were extracted from the fresh May and August MSW, respectively (Table S10). It is thus surprising that 6:2 FTOH was not measured in the reactor gas.

In contrast to the three MSW reactors that exhibited typical waste decomposition, as many as 11 volatile PFAS were detected in MSW-May a, which was inhibited, with 9 PFAS detected in the first gas sample on day 8 (Figure S11D). The 7:2-sFTOH was again detected on day 21, and 12:2 FTO was detected on day 61. Although multiple volatile PFAS were released from MSW-May a, the summed PFAS release was considerably lower than the PFAS release from the other MSW reactors (Table S10). MSW-May a generated considerably less CH_4_ than the other MSW reactors (Figure S5B). To the extent that PFAS release is governed by total CH_4_ release, which was the general trend in the high *F* reactors, the lower CH_4_ in MSW-May a explains its lower PFAS release.

## PFAS Release to Leachate

The release of PFAS to leachate was characterized by multiplying the maximum measured PFAS concentration by the volume of liquid in each reactor and results are presented in Figures S12–S16 and Table S11. PFAS release to leachate for the high *F* materials was up to 3 orders of magnitude higher than releases for the low *F* materials and MSW (Table S11). The dominant PFAS in leachate were PFBA, PFHxA, 5:3, and 6:2 FTCAs (Tables S11), and are likely due to the biodegradation of 6:2 FTOH in the test materials.^37^ Both 5:3 and 6:2 FTCAs and PFHxA were only detected in the high *F* materials, while PFBA was detected in all samples, with the exception of Popcorn Bags 2.

## Behavior of PIGE-*F* and Extractable PFAS

In addition to initial screening by PIGE, selected materials were subject to a methanol extraction prior to reactor loading and after completion of the decomposition cycle (Table S12). Methanol-extractable FTOHs from the food packaging materials represented <1% of total *F* as measured by PIGE (Table S12). Postdigestion, the total *F* computed from measured volatile PFAS (i.e., 6:2 FTOH) concentrations made up <1% of total PIGE-*F*, indicating the release of volatile PFAS to the gas phase accounts for a small portion of total *F* in the fresh high and low *F* packaging materials (Table S12). Further, the levels of total *F* calculated from the summed concentrations of FTOHs in the residual solids postdigestion were two-to-three orders-of-magnitude higher than the corresponding concentrations from the fresh materials, indicating that there was a net production of FTOH post digestion (Table S12). The formation of FTOH can be attributed to the hydrolysis and biodegradation of side-chain fluorinated polymers^36,37^ contained in the materials tested.

Finally, Table S13 shows the ratio of PFAS in leachate to PFAS released to the gas phase. Release of total *F* to leachate was either comparable or considerably higher (factor of 3–6000) than release to the gas. In contrast, work from Lin et al. on Florida landfills indicated that the mass of *F* leaving in leachate was comparable or less than the mass of *F* leaving in LFG (i.e., gas-phase).^22^ While release of the measured PIGE-*F* to the gas-phase in this reactor study was minimal, the FTOHs measured in the materials postdigestion will eventually degrade, likely to PFCAs;^46,47^ thus, explaining, in part, the presence of PFCAs in landfill leachate.^48,49^ In general, landfill leachate is treated at wastewater treatment plants that typically do not have processes for PFAS attenuation.^13^

## Implications

This is the first report of the release of volatile PFAS associated with single-use packaging materials to the gas phase during anaerobic decomposition. The FTOHs were the dominant PFAS released from the high *F* materials tested in this study, which is consistent with observations of the PFAS composition of landfill gas.^21^ Interestingly, although materials conformed to the EPA polymer guidelines of “no more than 2% measured monomer”, 6:2 FTOH emissions were nonetheless measurable.^50^

Depending on the landfill, some of the produced landfill gas is captured and burned in a flare, boiler or engine with unknown destruction efficiency, while some gas is released as a fugitive emission. We recently estimated that 48.8% of US landfill gas is collected and combusted.^51^ In ongoing work, we are developing a quantitative estimate of volatile PFAS release from US landfills.

In general, PFAS were released early in the decomposition cycle. The incubation temperature in this study (37 °C) is comparable or lower than that typical of landfills, although the temperature at waste burial is typically at ambient temperature and warms over time.^52^ Thus, the relationship between the temperature used in this study and PFAS release in a landfill is complex. The early release measured here suggests that PFAS will be released to the atmosphere as gas collection and control systems are rarely installed in landfills within a year of waste burial and fresh PFAS-containing waste is added over the life of the landfill. Nonetheless, the fact that PFAS have been reported in landfill gas indicates that release occurs over time. Subsequent work on PFAS release from landfills will evaluate whether waste age is correlated with PFAS concentrations in landfill gas. In a previous study of landfill leachate, 6 of 19 PFAS were lower in older samples.^13^

## Supporting Information Available

Additional tables and figures and a spreadsheet with all PFAS concentration and yield data are also included (XLSX)
analytical methods and additional discussion on the release of PFAS from the materials tested; also included are (1) data characterizing the tested materials and their decomposition; (2) a list of analytes; (3) a schematic of the reactor system; and (4) data supporting the discussion on the behavior of PIGE-*F* and extractable PFAS after decomposition (PDF)
