# Supplementary material for: Release of Volatile Per- and Polyfluoroalkyl Substances from Plant Fiber-Based Food Packaging and Municipal Solid Waste to Gas under Simulated Landfill Conditions
Source: Environ Sci Technol. 2024 Nov 19;58(48):21295–304. doi: 10.1021/acs.est.4c08544 (PMC11618979; doi:10.1021/acs.est.4c08544)

Supporting Information

Release of Volatile Per- and Polyfluoroalkyl Substances from Plant Fiber-based Food Packaging and Municipal Solid Waste to Gas Under Simulated Landfill Conditions

Yuemei Ye[1,#,*], Ivan A. Titaley[2], Mitchell L. Kim-Fu[2], Ansel R. Moll[2], Jennifer A. Field[2] and Morton A. Barlaz[1]

[1]Department of Civil, Construction, and Environmental Engineering, North Carolina State University, Campus Box 7908, Raleigh, North Carolina 27695-7908, United States

[2]Department of Environmental and Molecular Toxicology, Oregon State University, 1007 Agriculture & Life Sciences Building, Corvallis, Oregon 97331, United States

[#]Current Affiliation: Department of Chemistry, Lehman College, City University of New York, 250 Bedford Park Boulevard West, Bronx, New York 10468, United States

[*]Corresponding Author: yuemei.ye@lehman.cuny.edu

Number of pages: 39

Number of tables: 13

Number of Figure: 16

List of Tables

Table S1. Fluorine content of food packaging materials as screened by PIGE and GC-MS for total fluorine and 6:2 FTOH, respectively...S12

Table S2. Organic content and biochemical methane potential of materials tested in landfill simulation reactors...S14

Table S3. List of target volatile PFAS analytes for food packaging materials after methanol extraction and for gas sample analysis by TD-GC-MS...S15

Table S4. List of 17 suspect volatile PFAS analytes for gas sample analysis...........................S17

Table S5. List of 52 target ionic PFAS analytes in leachate using liquid chromatography tandem-mass spectrometry (LC-MS/MS) S18

Table S6. List of 21 target analytes in EPA 537M method used for ionic PFAS background check of the reactor system. Samples were analyzed using LC-MS/MS by SGS North America Inc. S21

Table S7. Mass of materials and volume of inoculum added to each reactor and the extent of cellulose and hemicellulose decomposition in each material.....................................S22

Table S8. Volatile PFAS released in Popcorn Bags 1 reactor after 27 days and Popcorn Bags 2 reactor at day 29 and day 203 (final day)..S23

Table S9. Measured 6:2 FTOH concentration ranges in the generated gas for each high F material ……………………………………………………………………………...S23

Table S10. Cumulative volatile PFAS yield in Low F and MSW reactors (ng/g) at the end of the monitoring period. S24

Table S11. Cumulative PFAS yield (ng/g) in leacahte for all reactors and the ratio of FTCA released in leachate to 6:2 FTOH released to gas phase. S25

Table S12. Profile of volatile PFAS and total F for select samples pre- and post-digestion......S26

Table S13. Summed PFAS as F released in leachate and ratio of leachate F to gas phase F.....S27

List of Figures

Figure S1. Schematic of the test reactor. **A.** 2L-glass reactor with flange connection part; **B.** drainage layer of pea gravel; **C.** #120 stainless steel woven wire mesh to reduce clogging of the reactor outlet; **D.** glass cap for the reactor with flange connection part; **E.** flange clamps to hold the two-pieces of glass together and keep the system sealed; a 245 EPDM gasket was placed between the two pieces of glass; **F.** stainless steel adjustable clamp; **G.** 10 mL Norm-Ject Luer lock polypropylene syringe; **H.** T-shape plastic 3-way barb fitting, polypropylene, 1/4" OD; **I.** Cole-Parmer female/male Luer hose barb adapter, 1/4" OD; **J.** McMaster quick-disconnect tube couplings, polysulfone, 1/4" OD; **K.** tubing flow control hose clamp, 3/4" OD 54; **L.** stainless steel quick-turn tube couplings, 1/4" OD; **M.** one-way Luer stopcock; **N.** tygon tube, 1/4" ID, flexible, clear, PVC; **O.** 10 L-foil gas bag (SKC. Inc.), **P.** 2L-Thermo Scientific™ Labtainer™ bioProcess container, with 3 Ports, MPC Insert, MPC Body, and Luer lock............................S28

Figure S2. Gas sampling system. **A**. 0.5 L-foil gas bag; **B.** check valve; **C.** 160 mL-serum bottle; **D.** H_2SO_4 aqueous solution (pH <3.7), 1 g/L methyl orange was added as a pH indicator (3.1-4.4); **E.** 18-gauge needles; **F.** rubber stopper to seal serum bottle. **G.** straight adapters, tube push-on barbed hose, 1/4" ID; **H**. Markes Universal gas sampling tube 1/4" OD (IS spiked TD tube); **I**. One-way Luer stopcock. S28

Figure S3. Measured PFAS concentrations in the Whatman #2 filter paper Control reactors. **A**. Control 1 reactor and **B**. Control 2 reactor S29

Figure S4. pH of all reactors: **A.** Control and Low F; **B.** MSW; **C.** High F (Natural Plates and Popcorn Bags), and **D.** High F (Compostable Bowls, Biodegradable Boxes, Bagasse Containers, and Eco-friendly Plates) S29

Figure S5. CH_4 yield in the reactors of **A.** Whatman #2 filter paper control; **B.** MSW; **C.** Popcorn bags, and **D.** High F (Compostable Bowls, Biodegradable Boxes, Bagasse Containers, and Eco-friendly Plates) S30

Figure S6. CH_4 generation rate (mL/day-dry g) of all reactors: **A.** Control and Low F; **B.** MSW; **C.** High F (Natural Plates and Popcorn Bags), and **D.** High F (Compostable Bowls, Biodegradable Boxes, Bagasse Containers, and Eco-friendly Plates) S31

Figure S7. The total fluorine concentration of 46 sampled materials (5 popcorn bags sampled in 2022 were not tested by PIGE)..S32

Figure S8. Volatile PFAS released from Popcorn Bags 1 reactor during the 27-day monitoring period. PFAS data are presented as a yield. As such, a flat line indicates that no PFAS was detected in a sample. The first gas sampling and detection day was day 12, and concentrations before day 12 are extrapolated S33

Figure S9. Time-dependent release of volatile PFAS to the gas phase in **A.** Natural Plates a and **B.** Natural Plates b. The summed PFAS and 6:2 FTOH lines overlap in both reactors..............S33

Figure S10. Volatile PFAS released from High F reactors. **A**. Compostable Bowls; **B.** Biodegradable Boxes; **C.** Bagasse Containers; **D.** Eco-friendly plates. The summed PFAS and 6:2 FTOH lines overlap in all reactors S34

Figure S11. Time-dependent release of volatile PFAS in **A**. Low F a, **B**. Low F b, **C**. MSW-August b, and **D**. MSW-May a reactor. The concentration of 7:2-sFTOH was first detected on day 74 for Low F a reactor and day 51 for Low F b reactor, 8:2 FTOH were first detected on day 43 in Low F b reactor, 10:2 FTOH were first detected on day 148 and 43 in Low F a and Low F b reactors; yields of these compounds before first detection were extrapolated. The summed PFAS and 7:2-sFTOH lines overlap in Figure S11C S35

Figure S12. PFAS concentration and relative abundance in leachate samples over time for the High F reactors: **A&B**. Compostable Bowls, **C&D**. Biodegradable Boxes, **E&F**. Bagasse Containers, **G&H**. Eco-friendly Plates, **I&J**. Popcorn Bags 1, and **K&L**. Popcorn Bags 2.......S36

Figure S13. PFAS concentration and relative abundance and CH_4 generation rate in the leachate samples over time for: **A&B**. High F Natural Plates a, **C&D**. High F Natural Plates b, and **E&F**. High F Natural Plates c. The discontinuous reporting of FTCAs concentrations in Natural Plates a could be due to the high LOQ of 5:3 FTCA (2500 ng/L) and 6:2 FTCA (2500 ng/L) S37

Figure S14. The PFAS concentration and relative abundance of each PFAS component in the leachate samples of **A&B.** Low F a **C&D.** Low F b S37

Figure S15. The PFAS concentration in the leachate samples of **A.** MSW-May a, **B.** MSW-May b, **C.** MSW-August a, and **D.** MSW-August b. In the case of PFOA, concentrations were 22 to 57% above the highest concentration in a control reactor..S38

Figure S16. **A.** Cumulative summed and individual ionic PFAS released to leachate from each reactor. **B.** The fraction of each PFAS released to the leachate S39

Materials and Methods

Particle Induced Gamma-ray Emission (PIGE) Spectroscopy. Briefly, each sample was cut into ~2.5×2.5 cm strips with methanol-rinsed scissors. Two gamma-ray characteristics generated at 110 keV and 197 keV due to the decay of the ^{19}F nucleus under continuous proton bombarding (3.4 MeV beam) were measured with a high-purity germanium detector to quantify the total fluorine concentration of the exposed strip. A calibration curve was established using a set of paper spiked with known concentrations of total fluorine. The final total fluorine of each sample was adjusted based on the actual density of each sample (**Equation 1**):

$$\text{Total fluorine (corrected, ng/g)} = \text{Total fluorine (measured, ng/g)} * \text{r}/800 \qquad (1)$$

(Total fluorine is described as PIGE-F in the manuscript.) The density was determined by cutting 4×4 cm strips of each collected food packaging material. Multiple layers of strips of each sample were packed together and the thickness was measured using Vernier calipers. The obtained thickness of the multilayered sample was then divided by the number of layers to get the thickness of a single-layer (d, m). The food packaging material density (ρ, kg/m^3) was calculated by dividing the average weight of a 4×4 cm strip by the strip's volume.

Inoculum Preparation. The inoculum used to initiate anaerobic decomposition was developed from a methane-producing consortia derived from decomposing MSW that has been grown on ground residential MSW for over 25 years.[1] To eliminate PFAS contamination in the inoculum, the culture was transferred to 85 mL biological growth medium containing PFAS-free Whatman #2 filter paper as the substrate in 160 mL serum bottles. After at least three transfers, the culture was used to inoculate larger vessels so to ultimately prepare ~20 L to be used to inoculate reactors. The presence of ionic PFAS in the methanogenic inoculum were analyzed using EPA 537M (**Table S6**) by a commercial lab (SGS). A trip control was sent with the samples to monitor any contamination during sample transport. No PFAS were detected in the inoculum or trip control.

Cellulose, Hemicellulose, and Klason lignin Analysis. About 1 g sample was extracted with 140 mL of toluene/ethanol (2:1, v/v) and then dried.[2] A known weight (~0.1- 0.3 g) of ground sample was subjected to a two-stage acid hydrolysis. Sugars (arabinose, galactose, glucose, mannose, and xylose) liberated from acid digestion were then analyzed by HPLC using an ICS

2500 pulsed electrochemical detector (Dionex, Sunnyvale, CA). Anhydro correction was used to convert glucose to cellulose and the other sugars to hemicellulose. Klason lignin was measured from the solids remaining after acid hydrolysis as the weight loss on ignition at 550 °C for 2 h. In the Klason lignin method, any organic material that is not soluble in the initial toluene/ethanol wash (which removes lipophilic extractives), and does not dissolve in sulfuric acid, will be counted as lignin.

Residual Solid Extraction for Rinsate Gas Chromatography-Mass Spectrometer (GC-MS) Analysis. Residual solids at the end of the reactor test in the reactors underwent an in-vial solid-liquid extraction for 11 volatile PFAS targets (Table S3) using methodology developed by Rewerts et al.[3] Briefly, 10 mg (± 4 mg) of residual solids were added to an autosampler vial with 1440 µL of methanol (99.8%, VWR, Radnor, PA). The vial was spiked with 60 µL of eight isotopically labeled internal standards for a final concentration of 100 pg/µL. Each vial was vortexed, sonicated for 10 minutes, and allowed to sit for at least 1 hour to settle solid particulates. If the concentration of the solid residual was beyond the calibration curve, the mass extracted was halved (5 mg (± 2 mg)). If the concentration was still above the calibration curve, a 1:20 (v/v) dilution of the 5 mg extract into methanol was done in a 2 mL centrifuge tube (VWR, Randor, PA) before being added to the autosampler vial to get the concentration of PFAS targets within the range of the calibration curve. Rinsate of the reactor did not undergo a pre-concentration step before analysis due to the potential loss of volatile PFAS. Therefore, to an autosampler vial with an insert, 288 µL of rinsate was added to 12 µL of eight isotopically labeled internal standards for a final concentration of 100 pg/µL and vortexed. All analyses for volatile PFAS were done using concurrent solvent recondensation-large volume injection GC-MS.[3]

GC-MS Analysis. For volatile PFAS analysis, 10 µL of extract was injected in splitless mode with an inlet temperature of 280 °C. A 4 mm i.d. single taper Topaz inlet liner with 15 mg deactivated quartz wool (Restek, Bellefonte, PA) was used. Helium was used as the carrier gas in a constant flow mode of 1 mL/min. Separations were performed using a deactivated, fused silica tubing capillary column (Agilent, 5 m × 0.53 mm i.d.) connected to an Rxi-624Sil MS capillary column (Restek, 30 m ×0.25 mm i.d., 1.40 µm film thickness). The GC oven temperature program was as follows: 50 °C for 2 min, ramped to 188 °C at a rate of 5 °C/min, then ramped to

300 °C at a rate of 15 °C/min for a total run time of 37.07 min. The Agilent 6890 GC was connected to an Agilent 5973N MS (Santa Clara, CA) that was operated in positive chemical ionization mode and in selected ion monitoring mode with methane as the reagent gas at a flow rate of 1 mL/min. Analyte concentrations for FTOHs, sFTOHs, FOSAs, and FOSEs were determined by a calibration curve with a minimum of 6 points with 1/x weighted linear regression or quadratic regression. All standards were prepared in the range of 1–2000 pg/µL. Continuing calibration verification standards (10 and 100 pg/µL) were analyzed after every five samples and concentrations were expected to fall within $\pm$ 30%. Method blanks and solvent blanks were analyzed to monitor potential carryover introduced during the experimental procedure; however, none was observed.[4]

Leachate Analytical Method. A solid phase extraction method was used to isolate the analytes from the leachate matrix before analysis. The frozen leachate samples were thawed and centrifuged to remove solids and spiked with an IS to a concentration of 800 ng/L. In preliminary work, a pilot study confirmed that the freezing and re-thawing steps did not negatively impact the analyzed ionic PFAS compounds. The spiked leachate was then loaded onto weak ion exchange cartridges (Waters Corp., 150 mg, 30 µm), which were pre-cleaned, pre-conditioned, and equilibrated with 4 mL 0.03% NH_4OH in methanol solution, 4 mL methanol, and 4 mL water before use. The samples were eluted with methanol and 0.03% NH_4OH methanol solution. Both eluents were collected for each sample. To ensure consistent sample composition for all analyzed leachate samples, the extracted samples were dried in a SpeedVac without heat and at the lowest pressure setting (0.1 Torr). The dried samples were stored in a -20°C freezer before being reconstituted with 2 mL of 90:10 water: methanol (*v/v*). Methanol was added first to reconstitute highly hydrophobic PFAS, then water was added to reconstitute the remaining PFAS to minimize PFAS loss.[5]

The leachate samples were analyzed with an Agilent 1290 LC coupled to a 6495c Agilent QqQ MS/MS using two separate methods: the first method consisted of a large panel of PFAS that has been previously published[5, 6] and the second method a FTCA only panel. An InfinityLab Poroshell HPH-C18 delay column (3.0 x 50 mm, 4µm; Agilent, Santa Clara, CA) was used for the large PFAS panel. Approximately 250 µL of leachate sample was injected into the instrument for analysis of 52 ionic PFAS analytes (Table S5). Sample preparation controls consisted of a

neat positive control (light and heavy calibration mixes spiked into distilled water [800 ng/L, PFAS panel; 3ng/mL, FTCA panel]) which was used to monitor extraction efficiency, and a negative neat control (spiked with only the heavy mix) to monitor whether contamination occurred during the SPE step. The instrument controls were a matrix matched extracted positive control [800 ng/L, PFAS panel; 3ng/mL, FTCA panel] to track instrument sensitivity over time and a negative control (IS only into matched matrix) to track potential carryover. Those controls were analyzed after the calibration curve and after every twenty unknown samples.

For the FTCA panel method, a 50 µL aliquot was injected onto a Kinetex F5 (2.1 x 100 mm, 100 Å; Phenomenex, Torrance, CA) analytical column at 50°C for separation. Aqueous (solvent A: water with 5% ACN and 0.1% formic acid) and organic (solvent B: ACN with 5% water and 0.1% formic acid) solvents were run at 500 µL/min using the following gradient: 0 min: 1% B, 0.5 min 50% B, 4 min: 99% B, 6 min: 99% B, 6.01 min: 1% B, & 7 min: 1%. Additionally, an InfinityLab Poroshell HPH-C18 delay column (3.0 x 50 mm, 4µm; Agilent, Santa Clara, CA) was installed in the flow path of the LC to delay any potential PFAS contamination in the HPLC solvent from interfering with the sample analysis.

The method detection limits were 2500 ng/L for 5:3 FTAC and 6:2 FTCA, and 1-2 ng/L for the other ionic PFAS analytes. A higher calibration range was needed for the FTCA panel due to the high background noise from the PFAS free matrix.

Results and Discussion

PFAS Release to Leachate.

Cumulative PFAS release to leachate is presented in **Figures S12 - S16**, and in **Table S11**.

Release of PFAS to Leachate from the High F Materials

High concentrations of 5:3 FTCA and 6:2 FTCA were detected in the leachate from most of the high F materials (**Figure S16**B). The high F materials also had the highest volatile PFAS yields (343-799 ng/g, dominated by 6:2 FTOH) (**Table 2**). Both 5:3 FTCA and 6:2 FTCA have been

reported as the principal biotransformation products of 6:2 FTOH in landfill leachate,[7] biosolids,[8] and river sediments.[9] While 6:2 FTOH was detected in the gas phase around day 10, FTCAs were detected in the leachate as early as day 33 (Compostable bowls) and as late as day 254 (Bagasse containers). The ratio of both 5:3 FTCA plus 6:2 FTCA released in leachate to the summed 6:2 FTOH released into the gas phase ranged from 42% to 198% in the Natural plates a-c, Compostable bowls, Biodegradable boxes, and Bagasse containers reactors (**Table S11**). This is consistent with the observation that 6:2 FTOH is released early and that some is converted to FTCAs. Of course, the FTOH released to the gas phase represents removal from a reactor and presumably, some FTOH was converted to FTCAs prior to its release in the reactor gas. The discontinuous presence of FTCAs in the Natural plates (**Figure S13**) could be due to the high LOQ of 5:3 FTCA (2500 ng/L) and 6:2 FTCA (2500 ng/L).

Both 5:3 FTCA and 6:2 FTCA were released from all high F reactors, except for the Popcorn bags and Eco-friendly plates. In the case of Popcorn bag 1, it is possible that monitoring was terminated prior to FTCA generation. Another possibility is that it was not detected due to the high LOQ. Surprisingly, FTCA was not detected in Popcorn bag 2 or Eco-friendly plates despite the extended monitoring time and high FTOH release. Perfluorohexanoic acid (PFHxA) was the dominant compound measured in the leachate from both Popcorn reactors as well as from the Eco-friendly plates. PFHxA has been reported as the primary impurity, degradant, and metabolite of the side-chain fluorinated polymers and fluorosurfactants due to the shift to short-chain fluorotelomer-based products.[10-13]

Release of PFAS to Leachate from the Low F and MSW Materials

Leachate PFAS release from the low F materials is summarized in **Figure S16** and **Table S11**. PFAS release is generally 2 orders of magnitude lower than that from the high F materials, which is consistent with the trend observed for volatile PFAS release. In contrast to the high F reactors, neither 6:2 FTCA or 5:3 FTCA were detected (**Table S11**). Instead, relatively low concentrations of PFBA, PFPeA, 6:2 FTS, and GenX were released to the leachate. Similarly, in the leachate from the three MSW reactors that exhibited typical anaerobic decomposition (MSW-May a, MSW-August a, and MSW-August b), small amounts of PFBA, PFPeA, PFHxA, 6:2 FTS, and GenX were released (**Table S11**). No FTCAs were detected in the MSW leachate although they are the dominant PFAS in landfill leachate (e.g., 5:3 FTCA, 7:3 FTCA).[14, 15] However, similar to landfill leachate, other short-chain PFAS that are significant in landfill leachate were detected (e.g., PFBA, PFBS, PFPeA, PFHxA).[15]

References

(1) Wang, Y.-S.; Byrd, C. S.; Barlaz, M. A. Anaerobic biodegradability of cellulose and hemicellulose in excavated refuse samples using a biochemical methane potential assay. *J. Ind. Microbiol. Biotech.* **1994**, *13* (3), 147-153.
(2) De la Cruz, F. B.; Yelle, D. J.; Gracz, H. S.; Barlaz, M. A. Chemical changes during anaerobic decomposition of hardwood, softwood, and old newsprint under mesophilic and thermophilic conditions. *J. Agric. Food Chem.* **2014**, *62* (27), 6362-6374.
(3) Rewerts, J. N.; Morré, J. T.; Massey Simonich, S. L.; Field, J. A. In-vial extraction large volume gas chromatography mass spectrometry for analysis of volatile PFASs on papers and textiles. *Environ. Sci. Technol.* **2018**, *52* (18), 10609-10616.
(4) Cahuas, L.; Titaley, I. A.; Field, J. A. Mass-labeled fluorotelomer alcohol fragmentation gives "false positive" for nonlabeled fluorotelomer alcohols with implications for consumer product analysis. *J. Am. Soc. Mass Spectrom.* **2022**, *33* (2), 399-403.
(5) Weed, R. A.; Boatman, A. K.; Enders, J. R. Recovery of per-and polyfluoroalkyl substances after solvent evaporation. *Environ. Sci.: Process. Impacts* **2022**, *24* (12), 2263-2271.
(6) Enders, J. R.; Weed, R. A.; Griffith, E. H.; Muddiman, D. C. Development and validation of a high resolving power absolute quantitative per-and polyfluoroalkyl substances method incorporating skyline data processing. *Rapid Commun. Mass Spectrom.* **2022**, *36* (11), e9295.

(7) Allred, B. M.; Lang, J. R.; Barlaz, M. A.; Field, J. A. Physical and biological release of poly- and perfluoroalkyl substances (PFASs) from municipal solid waste in anaerobic model landfill reactors. *Environ. Sci. Technol.* **2015**, *49* (13), 7648-7656.
(8) Zhang, S.; Szostek, B.; McCausland, P. K.; Wolstenholme, B. W.; Lu, X.; Wang, N.; Buck, R. C. 6: 2 and 8: 2 fluorotelomer alcohol anaerobic biotransformation in digester sludge from a WWTP under methanogenic conditions. *Environ. Sci. Technol.* **2013**, *47* (9), 4227-4235.
(9) Zhao, L.; Folsom, P. W.; Wolstenholme, B. W.; Sun, H.; Wang, N.; Buck, R. C. 6: 2 Fluorotelomer alcohol biotransformation in an aerobic river sediment system. *Chemosphere* **2013**, *90* (2), 203-209.
(10) Luz, A. L.; Anderson, J. K.; Goodrum, P.; Durda, J. Perfluorohexanoic acid toxicity, part I: development of a chronic human health toxicity value for use in risk assessment. *Regul. Toxicol. Pharmacol.* **2019**, *103*, 41-55.
(11) Wang, Z.; Cousins, I. T.; Scheringer, M.; Hungerbühler, K. Fluorinated alternatives to long-chain perfluoroalkyl carboxylic acids (PFCAs), perfluoroalkane sulfonic acids (PFSAs) and their potential precursors. *Environ. Int.* **2013**, *60*, 242-248.
(12) Scheringer, M.; Trier, X.; Cousins, I. T.; de Voogt, P.; Fletcher, T.; Wang, Z.; Webster, T. F. Helsingør statement on poly-and perfluorinated alkyl substances (PFASs). *Chemosphere* **2014**, *114*, 337-339.
(13) Wang, Z.; Cousins, I. T.; Scheringer, M.; Hungerbuehler, K. Hazard assessment of fluorinated alternatives to long-chain perfluoroalkyl acids (PFAAs) and their precursors: status quo, ongoing challenges and possible solutions. *Environ. Int.* **2015**, *75*, 172-179.
(14) Lang, J. R.; Allred, B. M.; Field, J. A.; Levis, J. W.; Barlaz, M. A. National estimate of per- and polyfluoroalkyl substance (PFAS) release to US municipal landfill leachate. *Environ. Sci. Technol.* **2017**, *51* (4), 2197-2205.
(15) Capozzi, S. L.; Leang, A. L.; Rodenburg, L. A.; Chandramouli, B.; Delistraty, D. A.; Carter, C. H. PFAS in municipal landfill leachate: occurrence, transformation, and sources. *Chemosphere* **2023**, *334*, 138924.

Table S1. Fluorine content of food packaging materials as screened by PIGE and GC-MS for total fluorine and 6:2 FTOH, respectively.

Material	Paper Density (kg/m^3)	Total fluorine conc. (PIGE-F)	Total fluorine conc. (PIGE F)-corrected based on density of paper[e]	Volatile PFAS (GC-MS)[a]	High or Low F Designation[c]
		(ng/g)	(ng/g)	6:2 FTOH[b] (ng/g)	
hamburger wrappers 1	750	6000	6400	NA	L
hamburger wrappers 2	1100	5000	2660	NA	L
hamburger wrappers 3	1000	2000	1600	NA	L
hamburger wrappers 4	1010	8000	6360	NA	L
restaurant burger fair bag	1100	11000	8010	NA	L
paper plates-1	790	<LOD	<LOD	NA	L
hot dog boxes	1310	3000	1830	NA	L
paper plates- 2	660	1000	1220	NA	L
microwavable popcorn bags a-2021	*1090*	*774000*	*571000*	*1150*	*H*
microwavable popcorn bags b-2021	1270	<LOD	<LOD	NA	L
microwavable popcorn bags c-2021	*1110*	*535000*	*386000*	*4730*	*H*
microwavable popcorn bags d-2021	*1060*	*1040000*	*1030000*	*1880*	*H*
microwavable popcorn bags e-2021[d]	*1140*	*999000*	*703000*	*89.2*	*H*
microwavable popcorn bags f-2021	*1060*	*1160000*	*869000*	*NA*	*H*
microwavable popcorn bags g-2021	*1090*	*900000*	*661000*	*1490*	*H*
microwavable popcorn bags a-2022	NA	NA	NA	NA	L
microwavable popcorn bags c-2022	*NA*	*NA*	*NA*	*8310*	*H*
microwavable popcorn bags d-2022	NA	NA	NA	NA	L
microwavable popcorn bags e-2022	NA	NA	NA	NA	L
microwavable popcorn bags f-2022	*NA*	*NA*	*NA*	*2470*	*H*
microwavable popcorn bags g-2022	NA	NA	NA	NA	L
compostable bowls	*650*	*1592000*	*1970000*	*3730*	*H*
microwave kraft containers	610	<LOD	<LOD	NA	L
concrete eco-friendly food cones	820	7000	6860	NA	L
unbleached parchment paper	850	25000	23600	NA	L
take-out boxes-1	760	43000	45400	NA	L
microwave takeout boxes	840	24000	22800	NA	L
eco-friendly food holder trays (sugar fiber is made with 100% non-toxic plant byproduct material)	770	62000	64800	NA	L
stand up ziploc bags	850	46000	43200	NA	L
disposable lunch bags	750	51000	54500	NA	L
biodegradable boxes	*730*	*1590000*	*1750000*	*3560*	*H*
grease-proof sturdy food trays	860	89000	82900	NA	L
eupako paper bags	800	107000	107000	NA	L
take-out boxes- 2	790	16000	16200	NA	L
bagasse containers (sugar cane residue)	500	1640000	2620000	3420	H
greaseproof paper french fry cups	1020	1000	780	NA	L
kraft compostable paper food cups	77	35000	361000	NA	L
food paper	2830	264000	74800	NA	L
disposable grease resistant kraft bags	250	12000	38400	NA	L

essentials patty paper	7940	<LOD	<LOD	NA	L
natural plates	*640*	*1160000*	*1440000*	*3780*	*H*
eco-friendly plates (sugar fiber is made with 100% non-toxic plant byproduct material)	*650*	*1480000*	*1810000*	*6410*	*H*
disposable french fry cups	820	9000	8760	NA	L
meat hugger natural freezer paper	702	2000	2280	NA	L
bamboo fiber cups	820	45000	43800	NA	L
grease resistant sandwich liners	780	300000	309000	NA	L

[a]Food packaging materials with 'L' designations were not analyzed using GC-MS.
[b]In the case of the 2022 popcorn bag samples, PIGE analyses were not available and the classification as High or Low F was based on the methanol extraction data alone.
[c]Food packaging materials with 'H' designations are *italicized.*
[d]popcorn bag e-2021 was classified as High F despite the relatively low 6:2 FTOH based on the high F as measured by PIGE (9.99×10^8 ng/g) and the possibility that popcorn bag e-2021 contained other volatile PFAS.
[e]The total F measured using PIGE does not account for the density differences between the samples and the standard. Therefore, the total F values used to help identify the high (H) and low (L) designations have been corrected to account for density differences (see eqn. 1).
<LOD = less than limit of detection.
NA = not analyzed.

Table S2. Organic content and biochemical methane potential of materials tested in landfill simulation reactors.

Reactor groups	Substrates in reactor	BMP (mL CH_4/g paper)	Cellulose (%)	Hemicellulose (%)	Lignin (%)
**High F**	Popcorn Bags 1 - 6 brands microwavable popcorn bags collected in 2021	327	65	21	10.93
	Popcorn Bags 2 - 2 brands microwavable popcorn bags collected in 2022	328	73	11	2.69
	compostable bowls	319	62	26	11.33
	biodegradable boxes	362	62	25	10.97
	bagasse containers	294	71	20	8.9
	natural plates	313	68	22	9.98
	eco-friendly plates	342	66	22	10.74
**Low F**	mixed paper plates, eco-friendly food trays, and poly coated freezer paper	200	58	14	27
**Control**	Whatman #2 filter paper	299	97	1	0.92
**MSW-May**	Fresh residential solid waste from Raleigh in May 2022	173	31	7	23.12
**MSW-August**	Fresh residential solid waste from Raleigh in August 2022	129	34	6	15.98

Table S3. List of target volatile PFAS analytes for food packaging materials after methanol extraction and for gas sample analysis by TD-GC-MS.[a]

CAS no.	Analyte	Abbreviation	Formula	Vendor	Molecular weight (a.m.u.)	Quantifier ion (m/z)	Qualifier ion (m/z)	Surrogate standard	Liquid Extract Analysis[b]	Gas Phase Analysis[c]
2043-47-2	4:2 fluorotelomer alcohol	4:2 FTOH	$C_6H_5F_9O$	Wellington	264	265	227	MFBET	×	×
647-42-7	6:2 fluorotelomer alcohol	6:2 FTOH	$C_8H_5F_{13}O$	Wellington	364	365	327	MFHET	×	×
678-39-7	8:2 fluorotelomer alcohol	8:2 FTOH	$C_{10}H_5F_{17}O$	Wellington	464	465	427	M2FOET	×	×
865-86-1	10:2 fluorotelomer alcohol	10:2 FTOH	$C_{12}H_5F_{21}O$	Wellington	564	565	527	MFDET	×	×
39239-77-5	12:2 fluorotelomer alcohol	12:2 FTOH	$C_{14}H_5F_{25}O$	SynQuest	664	665	627	MFDET	×	×
31506-32-8	*N*-methyl perfluorooctane sulfonamide	MeFOSA	$C_9H_4NO_2SF_{17}$	Wellington	513	514	-	*d*$_3$-N-MeFOSA-M	×	×
4151-50-2	*N*-ethyl perfluorooctane sulfonamide	EtFOSA	$C_{10}H_6NO_2SF_{17}$	Wellington	527	528	-	*d*$_5$-N-EtFOSA-M	×	×
24448-09-7	*N*-methyl perfluorooctane sulfonamidoethanol	MeFOSE	$C_{11}H_8NO_3SF_{17}$	Wellington	557	540	558	*d*$_7$-N-MeFOSE-M	×	×
1691-99-2	*N*-ethyl perfluorooctane sulfonamidoethanol	EtFOSE	$C_{12}H_{10}NO_3SF_{17}$	Wellington	571	554	572	*d*$_9$-N-EtFOSE-M	×	×
17527-29-6	6:2 fluorotelomer acrylate	6:2 FTAc	$C_{11}H_7F_{13}O_2$	SynQuest	418	419	-	*d*$_5$-6:2 FTMAc		×
27905-45-9	8:2 fluorotelomer acrylate	8:2 FTAc	$C_{13}H_7F_{17}O_2$	Wellington	518	519	-	*d*$_5$-6:2 FTMAc		×
17741-60-5	10:2 fluorotelomer acrylate	10:2 FTAc	$C_{15}H_7F_{21}O_2$	Wellington	618	619	-	*d*$_5$-6:2 FTMAc		×
2144-53-8	6:2 fluorotelomer methylacrylate	6:2 FTMAc	$C_{12}H_9F_{13}O_2$	SynQuest	432	433	461	*d*$_5$-6:2 FTMAc		×
1996-88-9	8:2 fluorotelomer methylacrylate	8:2 FTMAc	$C_{14}H_9F_{17}O_2$	SynQuest	532	533	561	*d*$_5$-6:2 FTMAc		×
25291-17-2	6:2 fluorotelomer olefin	6:2 FTO	$C_8H_3F_{13}$	SynQuest	346	327	-	*d*$_5$-6:2 FTMAc		×
21652-58-4	8:2 fluorotelomer olefin	8:2 FTO	$C_{10}H_3F_{17}$	Matrix	446	427	-	*d*$_5$-6:2 FTMAc		×
30389-25-4	10:2 fluorotelomer olefin	10:2 FTO	$C_{12}H_3F_{21}$	Matrix	546	527	-	*d*$_5$-6:2 FTMAc		×
67103-05-3	12:2 fluorotelomer olefin	12:2 FTO	$C_{14}H_3F_{25}$	SynQuest	646	627	-	*d*$_5$-6:2 FTMAc		×
355-43-1	Perfluorohexyl iodide	PFHxI	$C_6F_{13}I$	SynQuest	446	319	427	7Me-6:2 FTI		×
507-63-1	Perfluorooctyl iodide	PFOI	$C_8F_{17}I$	SynQuest	546	419	527	7Me-6:2 FTI		×
423-62-1	Perfluorodecyl iodide	PFDI	$C_{10}F_{21}I$	SynQuest	646	519	627	7Me-6:2 FTI		×
2043-55-2	4:2 fluorotelomer iodide	4:2 FTI	$C_6H_4F_9I$	SynQuest	374	355	403	7Me-6:2 FTI		×
2043-57-4	6:2 fluorotelomer iodide	6:2 FTI	$C_8H_4F_{13}I$	SynQuest	474	455	503	7Me-6:2 FTI		×
2043-53-0	8:2 fluorotelomer iodide	8:2 FTI	$C_{10}H_4F_{17}I$	SynQuest	574	555	603	7Me-6:2 FTI		×
2043-54-1	10:2 fluorotelomer iodide	10:2 FTI	$C_{12}H_4F_{21}I$	SynQuest	674	655	703	7Me-6:2 FTI		×

24015-83-6	7:2 secondary fluorotelomer alcohol†	7:2-sFTOH	$C_9H_5F_{15}O$	Wellington	414	377	415	MFHET	×	
914637-05-1	5:2 secondary fluorotelomer alcohol†	5:2-sFTOH	$C_7H_5F_{11}O$	Wellington	314	277	315	MFBET	×	

[a]The 5:2-sFTOH and 7:2-sFTOH were targets in the liquid extracts because target standards were acquired after gas phase analyses were complete. However, they were suspects for gas phase analyses.

[b,c] All PFAS have "×" in the same row is included in this method.

Wellington: Wellington Laboratories (Guelph, ON), SynQuest: SynQuest Laboratories (Alachua, FL), Matrix: Matrix Scientific (Columbia, SC), a.m.u.: atomic mass unit.

Table S4. List of 17 suspect volatile PFAS analytes for gas sample analysis.

CAS no.	Analyte	Abbreviation	Formula	Molecular weight (a.m.u.)	Quantifier ion (*m/z*)	Qualifier ion (*m/z*)	Target PFAS calibration curve used for semi quantification
60699-51-6	14:2 fluorotelomer alcohol	14:2 FTOH	$C_{16}H_5F_{29}O$	764	765	727	10:2 FTOH
68298-12-4	*N*-methyl perfluorobutane sulfonamidoethanol	MeFBSA	$C_5H_4NO_2SF_9$	313	314	-	MeFOSA
68259-15-4	*N*-methyl perfluorohexane sulfonamidoethanol	MeFHxSA	$C_7H_4NO_2SF_{13}$	413	414	-	MeFOSA
Not found	*N*-methyl perfluoropropane sulfonamidoethanol	MeFPrSE	$C_6H_8NO_3SF_7$	307	290	308	MeFOSE
34454-97-2	*N*-methyl perfluorobutane sulfonamidoethanol	MeFBSE	$C_7H_8NO_3SF_9$	357	340	358	MeFOSE
68555-74-8	*N*-methyl perfluoropentane sulfonamidoethanol	MeFPeSE	$C_8H_8NO_3SF_{11}$	407	390	408	MeFOSE
68555-75-9	*N*-methyl perfluorohexane sulfonamidoethanol	MeFHxSE	$C_9H_8NO_3SF_{13}$	457	440	458	MeFOSE
68555-76-0	*N*-methyl perfluoroheptane sulfonamidoethanol	MeFHpSE	$C_{10}H_8NO_3SF_{17}$	507	490	508	MeFOSE
Not found	*N*-ethyl perfluoroethane sulfonamidoethanol	EtFEtSE	$C_{12}H_{10}NO_3SF_{17}$	271	254	272	EtFOSE
Not found	*N*-ethyl perfluoropropane sulfonamidoethanol	EtFPrSE	$C_{12}H_{10}NO_3SF_{17}$	321	304	322	EtFOSE
34454-97-2	*N*-ethyl perfluorobutane sulfonamidoethanol	EtFBSE	$C_{12}H_{10}NO_3SF_{17}$	371	354	372	EtFOSE
68555-74-8	*N*-ethyl perfluoropentane sulfonamidoethanol	EtFPeSE	$C_{12}H_{10}NO_3SF_{17}$	421	404	422	EtFOSE
68555-75-9	*N*-ethyl perfluorohexane sulfonamidoethanol	EtFHxSE	$C_{12}H_{10}NO_3SF_{17}$	471	454	472	EtFOSE
68555-76-0	*N*-ethyl perfluoroheptane sulfonamidoethanol	EtFHpSE	$C_{12}H_{10}NO_3SF_{17}$	521	504	522	EtFOSE
24015-83-6	7:2 secondary fluorotelomer alcohol	7:2-sFTOH	$C_9H_5F_{15}O$	414	415	395	8:2 FTOH
914637-05-1	5:2 secondary fluorotelomer alcohol	5:2-sFTOH	$C_7H_5F_{11}O$	314	315	295	6:2 FTOH
375-14-4	3:2 secondary fluorotelomer alcohol	3:2-sFTOH	$C_5H_5F_7O$	214	215	195	4:2 FTOH

Table S5. List of 52 target ionic PFAS analytes in leachate using liquid chromatography tandem-mass spectrometry (LC-MS/MS).[a,b]

CAS #	Analyte	Abbreviation	Neutral Molecular Formula	Matching IS[c]	[d]LOQ (ng/L)	% Recovery ± SD[e]
**Per and Polyfluoroalkyl carboxylic acid (PFCA)**						
375-22-4	Perfluorobutanoic acid	PFBA	$C_4HF_7O_2$	×	10	96 ± 9
2706-90-3	Perfluoropentanoic acid	PFPeA	$C_5HF_9O_2$	×	5	103 ± 12
307-24-4	Perfluorohexanoic acid	PFHxA	$C_6HF_{11}O_2$	×	2	88 ± 10
375-85-9	Perfluoroheptanoic acid	PFHpA	$C_7HF_{13}O_2$	×	5	86 ± 11
335-67-1	Perfluorooctanoic acid	PFOA	$C_8HF_{15}O_2$	×		64 ± 11
375-95-1	Perfluorononanoic acid	PFNA	$C_9HF_{17}O_2$	×	100	88 ± 11
335-76-2	Perfluorodecanoic acid	PFDA	$C_{10}HF_{19}O_2$	×	100	86 ± 10
2058-94-8	Perfluoroundecanoic acid	PFUnDA	$CF_3(CF_2)_9CO_2H$	×		
307-55-1	Perfluorododecanoic acid	PFDoDA	$C_{12}HF_{23}O_2$	×	100	91 ± 7
72629-94-8	Perfluorotridecanoic acid	PFTrDA	$C_{13}HF_{25}O_2$			
376-06-7	Perfluorotetradecanoic acid	PFTeDA	$CF_3(CF_2)_{12}CO_2H$	×	100	94 ± 12
67905-19-5	Perfluorohexadecanoic acid	PFHxDA	$C_{16}HF_{31}O_2$	×		
16517-11-6	Perfluorooctadecanoic acid	PFODA	$C_{18}HF_{35}O_2$			
**Per and Polyfluoroalkyl sulfonic acids (PFSA)**						
375-73-5	Perfluorobutanesulfonic acid	PFBS	$C_4HF_9O_3S$	×	10	95 ± 16
2706-91-4	Perfluoropentanesulfonic acid	PFPeS	$C_5HF_{11}O_3S$			
355-46-4	Perfluorohexanesulfonic acid	PFHxS	$C_6HF_{13}O_3S$	×	10	87 ± 8
375-92-8	Perfluoroheptanesulfonic acid	PFHpS	$C_7HF_{15}O_3S$			
1763-23-1	Perfluorooctanesulfonic acid	PFOS	$C_8HF_{17}O_3S$	×	10	92 ± 10
68259-12-1	Perfluorononanesulfonic acid	PFNS	$C_9HF_{19}O_3S$			
2806-15-7	Perfluorodecanesulfonic acid	PFDS	$C_{10}F_{21}NaO_3S$			
**Per and Polyfluoroether carboxylic acid (PFECA)**						
674-13-5	Perfluoro-2-methoxyacetic acid	PFMOAA	$C_3HF_5O_3$			
267239-61-2	Perfluoro-2-ethoxypropanoic acid	PEPA	$C_5HF_9O_3$			
39492-89-2	Perfluoro-3,5,7-trioxaoctanoic acid	PFO3OA	$C_5HF_9O_5$			
13252-13-6	Perfluoro-2-propoxypropanoic acid	Gen-X	$C_6HF_{11}O_3$	×	10	83 ± 10
39492-90-5	Perfluoro-3,5,7,9-butaoxadecanoic acid	PFO4DA	$C_5HF_9O_5$			
39492-91-6	Perfluoro-3,5,7,9,11-pentaoxadodecanoic acid	PFO5DoDA	$C_7HF_{13}O_7$			
773804-62-9	Perfluoroethoxsypropanoic acid	Hydro-EVE	$C_8H_2F_{14}O_4$			
919005-14-4	4,8-Dioxa-3H-perfluorononanoic acid	NaDONA	$C_7H_2F_{12}O_4$			
**Per and Polyfluoroether sulfonic acid (PFESA)**						

29311-67-9	Perfluoro-3,6-dioxa-4-methyl-7-octene-1-sulfonic acid	Nafion byproduct 1	$C_7HF_{13}O_5S$			
749836-20-2	Perfluoro-2-{[perfluoro-3-(perfluoroethoxy)-2-propanyl]oxy}ethanesulfonic acid	Nafion by-product 2	$C_7H_2F_{14}O_5S$			
801209-99-4	1,1,2,2-tetrafluoro-2-(1,2,2,2-tetrafluoroethoxy)ethanesulfonic acid	NVHOS	$C_4H_2F_8O_4S$			
73606-19-6	9-chlorohexadecafluoro-3-oxanonane-1-sulfonate	F53 Major	$C_8ClF_{16}KO_4S$			
83329-89-9	11-chloroeicosafluoro-3-oxaundecane-1-sulfonate	F53 Minor	$C_{10}ClF_{20}KO_4S$			
**Per and Polyfluoroalkyl sulfonamides (PFSAm)**						
30334-69-1	Perfluorobutane sulfonamide	PFBSA	$C_4H_2F_9NO_2S$			
41997-13-1	Perfluorohexane sulfonamide	PFHxSA	$C_6H_2F_{13}NO_2S$			
2355-31-9	N-methyl perfluorooctanesulfonamidoacetic acid	nMeFOSAA	$C_{11}H_6F_{17}NO_4S$	×		
2991-50-6	N-ethyl perfluorooctanesulfonamidoacetic acid	nEtFOSAA	$C_{12}H_8F_{17}NO_4S$	×		
754-91-6	Perfluorooctane sulfonamide	PFOSA	$C_8H_2F_{17}NO_2S$	×	1000	103 ± 12
31506-32-8	N-Methylperfluorooctanesulfonamide	N-MeFOSA	$C_9H_4F_{17}NO_2S$	×		
**Fluorotelomer carboxylic acids (FTCA)**						
914637-49-3	3-Perfluoropentyl-propanoic acid	5:3 FTCA	$C_8H_5F_{11}O_2$		2500	101 ± 7
53826-12-3	2-Perfluorohexyl-ethanoic acid	6:2 FTCA	$C_8H_3F_{13}O_2$	×	2500	83 ± 3
27854-31-5	2-Perfluorooctyl-ethanoic acid	8:2 FTCA	$C_{10}H_3F_{17}O_2$	×	2500	91 ± 7
812-70-4	3-Perfluoroheptyl propanoic acid	7:3 FTCA	$C_{10}H_5F_{15}O_2$		2500	
70887-88-6	2H-Perfluoro-2-octenoic acid	6:2 FTUCA	$C_8H_2F_{12}O_2$	×	2500	93 ± 1
70887-84-2	2H-Perfluoro-2-decenoic acid	8:2 FTUCA	$C_8H_2F_{12}O_2$	×	2500	106 ± 2
**Fluorotelomer sulfonic acids (FTS)**						
757124-72-4	4:2 Fluorotelomer sulfonic acid	4:2 FTS	$C_6H_5F_9O_3S$	×	1.6	82 ± 9
27619-97-2	6:2 Fluorotelomer sulfonic acid	6:2 FTS	$C_8H_5F_{13}O_3S$	×	5	87 ± 12
39108-34-4	8:2 Fluorotelomer sulfonic acid	8:2 FTS	$C_{10}H_5F_{17}O_3S$	×	10	91 ± 13
120226-60-0	10:2 Fluorotelomer sulfonic acid	10:2 FTS	$C_{12}H_5F_{21}O_3S$	×	5	89 ± 16
**Zwitterionic**						
50598-28-2	N-(3-dimethylaminopropan-1-yl)perfluoro-1-hexane-sulfonamide	N-AP-FHxSA	$C_{11}H_{13}F_{13}N_2O_2S$			

38850-51-0	N-[3-(perfluoro-1-hexanesulfonamido)propan-1-yl]-N,N,N-trimethylammonium	N-TAmP-FHxSA	$C_{12}H_{15}F_{13}N_2O_2S$
34455-29-3	6:2 Fluorotelomer sulfonamide betaine	N-CMAmP-62FOSA (62 FTAB)	$C_{15}H_{19}F_{13}N2O_4S$

[a]Included in the Fluoryx Mix.
[b]The green highlighted compounds are within the range of 70 to 130%. Uncolored compounds did not pass the data quality control. 24 out of 52 analytes passed the data quality control, and average % recovery and standard deviation were added to these compounds.
[c]All PFAS with "×" in the same row have match IS in the method.
[d]LOQ: Limit of quantitation.
[e]Average of 5 standard additions.

Table S6. List of 21 target analytes in EPA 537M method used for ionic PFAS background check of the reactor system. Samples were analyzed using LC-MS/MS by SGS North America Inc.

CAS no.	Analyte	Acronym	Neutral Molecular Formula	RL[a] (µg/L)	MDL[b] (µg/L)
**Perfluoroalkylcarboxylic Acids (PFCA)**					
375-22-4	Perfluorobutanoic acid	PFBA	$C_4HF_7O_2$	0.0080	0.0040
2706-90-3	Perfluoropentanoic acid	PFPeA	$C_5HF_9O_2$	0.0040	0.0020
307-24-4	Perfluorohexanoic acid	PFHxA	$C_6HF_{11}O_2$	0.0040	0.0020
375-85-9	Perfluoroheptanoic acid	PFHpA	$C_7HF_{13}O_2$	0.0040	0.0020
335-67-1	Perfluorooctanoic acid	PFOA	$C_8HF_{15}O_2$	0.0040	0.0020
375-95-1	Perfluorononanoic acid	PFNA	$C_9HF_{17}O_2$	0.0040	0.0020
335-76-2	Perfluorodecanoic acid	PFDA	$C_{10}HF_{19}O_2$	0.0040	0.0020
2058-94-8	Perfluoroundecanoic acid	PFAA	$C_{11}HF_{21}O_2$	0.0040	0.0020
307-55-1	Perfluorododecanoic acid	PFDoA	$C_{12}HF_{23}O_2$	0.0040	0.0020
72629-94-8	Perfluorotridecanoic acid	PFTrDA	$C_{13}HF_{25}O_2$	0.0040	0.0020
376-06-7	Perfluorotetradecanoic acid	PFTA	$C_{14}HF_{27}O_2$	0.0040	0.0020
**Perfluoroalkylesulfonic Acids**					
375-73-5	Perfluorobutanesulfonic acid	PFBS	$C_4HF_9O_3S$	0.0040	0.0020
355-46-4	Perfluorohexanesulfonic acid	PFHxS	$C_6HF_{13}O_3S$	0.0040	0.0020
1763-23-1	Perfluorooctanesulfonic acid	PFOS	$C_8HF_{17}O_3S$	0.0040	0.0020
**Perfluorooctanesulfonamides**					
754-91-6	Perfluorooctanesulfonamide	PFOSA	$C_8H_2F_{17}NO_2S$	0.0080	0.0040
**Perfluorooctanesulfonamidoacetic Acids**					
2355-31-9	N-Methylperfluorooctanesulfonamidoacetic acid	MeFOSAA	$C_{11}H_6F_{17}NO_4S$	0.0080	0.0040
2991-50-6	N-ethyl perfluorooctane sulfonamido acetic acid	EtFOSAA	$C_{12}H_8F_{17}NO_4S$	0.0080	0.0040
**Fluorotelomer Sulfonates**					
757124-72-4	4:2 Fluorotelomer sulfonate	4:2 FTS	$C_6H_5F_9O_3S$	0.016	0.0040
27619-97-2	6:2 Fluorotelomer sulfonate	6:2 FTS	$C_8H_5F_{13}O_3S$	0.016	0.0040
39108-34-4	8:2 Fluorotelomer sulfonate	8:2 FTS	$C_{10}H_5F_{17}O_3S$	0.016	0.0040
**Next Generation PFAS Analytes**					
13252-13-6	Hexafluoropropylene oxide dimer acid	HFPO-DA (GenX)	$C_6HF_{11}O_3$	0.0080	0.0040

[a]RL: Reporting Limit
[b]MDL: Method Detection Limit

Table S7. Mass of materials and volume of inoculum added to each reactor and the extent of cellulose and hemicellulose decomposition in each material.[a]

Reactor Information			Fresh Material				After Decomposition			Decomposition Extent (%)	
Reactor Groups	Substrates content	Volume of Inoculum (mL)	Mass of dried substrate (g)	Cellulose (%)	Hemicellulose (%)	Extractives (%)	Mass of dried substrate (g)	Cellulose (%)	Hemicellulose (%)	Cellulose Loss (%)	Hemicellulose Loss (%)
High F	natural plates	1900	100.2	67.86	22.12	0.04	10.67	8.15	2.80	98.72	98.65
		1900	100				46.39	70.19	10.35	52.02	78.29
		1900	100.3				26.05	9.99	3.00	96.18	96.48
	Popcorn Bags 1	1900	100.3	61.97	10.19	3.05		NA		NA	
	compostable bowls	1900	100.4	61.7	25.61	1.36	6.37	Missing	Missing	Missing	Missing
	biodegradable boxes	1900	99.9	62.36	25.09	1.58	19.02	5.12	1.9	98.44	98.56
	bagasse containers	1900	100.5	71	19.89	0.21	35.52				
	eco-friendly plates	1900	99.9	66.33	22.02	0.91	18.04	53.1	10.76	85.54	91.18
	Popcorn Bags 2	1800	100.4	65.3	7.63	11.87	22.8	27.19	4.57	86.75	86.06
				59.54	10.98	16.09					
Low F	Low F a	1500	101	57.62	14.00	1.58	37.54	27.69	10.09	82.14	73.22
	Low F b	1500	100.2				17.13	20.28	7.94	104.73	90.31
Control	Control 1	1503	100.1	97.04	1.14	17.98	6.52	40.52	0.86	97.28	95.09
	Control 2	1900	100.06					0		100.00	100.00
MSW	MSW-May a	1900	363.2	31.44	6.91	16.75		NA		NA	
	MSW-May b	1500	164.2								
	MSW-August a	1700	284.1	34.49	6.48	14.34					
	MSW-August b	1500	140.6								

[a]Decomposition is 1 minus the mass of cellulose or hemicellulose recovered at the end of the decomposition cycle divided by the initial mass.

Table S8. Volatile PFAS released in Popcorn Bags 1 reactor after 27 days and Popcorn Bags 2 reactor at day 29 and day 203 (final day).

PFAS yield (ng/g bag)	Popcorn bag 1 day 27	Popcorn bag 2-day 29	Popcorn bag 2-day 203
4:2 FTOH	0.00213	0	0
6:2 FTOH	14.56	52.8	99.5
8:2 FTOH	0.0062	0	0
10:2 FTOH	0.025	0	0
MeFOSA	0.0045	0	0
EtFOSA	0.0079	0	0
6:2 FTAc	0.00044	0	0
8:2 FTAc	0.038	0	0
10:2 FTAc	0.0068	0	0
8:2 FTMAc	0.011	0	0
6:2 FTO	0.84	0	0.0017
8:2 FTO	0.0055	0	0
10:2 FTO	0.0016	0	0
12:2 FTO	0.003	0	0.011
PFHxI	4.7	0	0
PFOI	0.081	0	0
4:2 FTI	0.045	0	0
6:2 FTI	0.0083	0	0
7:2-sFTOH	0.083	0	0

Table S9. Measured 6:2 FTOH concentration ranges in the generated gas for each high F material.

Reactors	Min (ng/L gas)	Max (ng/L gas)
Popcorn bags 1	0	1220
Popcorn bags 2	0	457
Compostable Bowls	2	5433
Biodegradable Boxes	250	2190
Bagasse Containers	101	602
Eco-friendly Plates	284	675
Natural Plates a	<LOD	5630
Natural Plates b	<LOD	3280
Natural Plates c	127	3570

Table S10. Cumulative volatile PFAS yield in Low F and MSW reactors (ng/g) at the end of the monitoring period.[a]

PFAS (ng/g)	Reactors					
	MSW-May a	MSW-May b	MSW-August a	MSW-August b	Low F	
					a	b
6:2 FTOH	<LOQ	<LOQ	<LOQ	<LOQ	<LOQ	0.0066
**6:2 FTOH**[b]	**14000**		**8000**		**NA**	
8:2 FTOH	<LOQ	<LOQ	<LOQ	<LOQ	<LOQ	0.0033
10:2 FTOH	<LOQ	<LOQ	<LOQ	0.013	0.02	0.0045
7:2-sFTOH	0.034	0.62	0.1	0.135	0.07	0.4
ΣPFAS	0.078	0.62	0.1	0.148	0.09	0.41

[a]if a compound is LOD in every case, then it was not included.

[b]6:2 FTOH in ground MSW material prior to testing.

Table S11. Cumulative PFAS yield (ng/g) in leachte for all reactors and the ratio of FTCA released in leachate to 6:2 FTOH released to gas phase.

Reactor groups	PFBA	PFPeA	PFHxA	PFHpA	PFOA[b]	4:2 FTS	6:2 FTS	GenX	5:3 FTCA	6:2 FTCA	Summed FTCAs	Summed PFAS (Leachate)	Summed PFAS (gas)	Summed FTCAs/6:2 FTOH in gas phase
high F-Natural Plates (a,b,c)	2.64	0.906	9.83	0.245		0.034			115	220	335	340	799	0.42
	4.71	0.228	6.2						410	142	553	561	487	1.13
	10.1	0.314	11.4						342	130	472	445	556	0.85
high F-Compostable Bowls	2.77	1.3	14.3	0.07				0.01	225	154	379	279	343	1.11
high F-Biodegradable Boxes	2.29	0.173	4.7						176	93	269	276	484	0.57
high F-Bagasse Containers	3.07	1.45	11.9	1.13				0.045	182	58	240	257	125	1.98
high F-Eco-friendly Plates	4.98	1.47	18.3	1.1			0.098	0.001				25.6	62	
high F-Popcorn Bag 1	0.009	0	0.757	0.203								0.96	20.4	
high F-Popcorn Bag 2	<LOD		0.862	0.648			0.029		[a]<LOQ	[a]< LOQ		15.9	99.7	
Low F-mixed paper plates, eco-friendly food trays, and polycoated freezer paper (A, B)	0.793	0.284										1.02	0.09	
	0.254	0.32					0.116	0.08				0.488	0.41	
MSW-May (a, b)	3.57	0.378	0.095		0.074		0.057	0.029				4.09	0.078	
	0.129	0.113	0.003				0.04					0.197	0.62	
MSW-August (a, b)	0.212	0.314										0.525	0.1	
		0.335						0.004				0.335	0.148	

[a]LOQ is 2500 ng/L.

[b]In the case of PFOA, concentrations were 22 to 57% above the highest concentration in a control reactor.

All results <LOD are left empty.

Table S12. Profile of volatile PFAS and total F for select samples pre- and post-digestion.

	Pre-digestion				*Post-digestion*						
**Reactor group**	**Total F from PIGE (ng/g)**	**Methanol extracted Neutral PFAS (6:2 FTOH) - (ng/dry g)**	**Methanol extracted Total F (ng)**	**Methanol extracted/PIGE Total F (%)**	**Methanol extracted Neutral PFAS (6:2 FTOH) in residual solid - (ng/wet g)**	**Methanol extracted Total F of neutral PFAS in residual solid (ng)**	**Summed F of neutral PFAS adsorbed on reactor and gasket (ng)**	**Summed volatile PFAS released in gas (ng)**	**Summed F on reactor and gasket/Total F of neutral PFAS released in gas (%)**	**Summed F of all gas phase neutral PFAS/Total F from PIGE (%)**	**Summed F in the residual solid/Total F in fresh material methanol extract (%)**
Low F-mixed paper plates, eco-friendly food trays, and polycoated freezer paper	<LOD[a]	NA[b]	NA	NC[c]	<LOD	NC	<LOD	9	NC	0	NC
	2280[d]		NA	NC	<LOD	NC	<LOD	41	NC	0.002	NC
	64800[d]										
high F-Natural Plates (a,b,c)	1590000	3560	241000	0.15	20000	1850000	<LOD	80000	NC	0.050	767
			240000	0.15	17000	2393000	464	48700	1.4	0.031	996
			241000	0.15	65000	5950000	457	55700	1.2	0.035	2470
high F-Compostable Bowls	1970000	3730	253000	0.13	92000	5330000	887	34400	3.8	0.018	2110
high F-Biodegradable Boxes	1750000	3420	231000	0.13	92000	8370000	<LOD	48300	NC	0.018	3630
high F-Bagasse Containers	2610000	3780	256000	0.10	160000	17300000	745	12500	8.8	0.005	6750
high F-Eco-friendly Plates	1810000	6410	432000	0.24	130000	18300000	327	6200	7.8	0.004	4230
High F-Popcorn Bags	NA	8310	563000	NC	2800	459000	47	10000	68.9	NC	84.9
	NA	2470	167000	NC							
MSW-May	NA	14000	3430000	NC	NA	NA	NA	28	NC	NC	NC
			1550000		NA		NA	102	NC	NC	NC
MSW-August	NA	8000	1530000	NC	NA	NA	NA	31	NC	NC	NC
			759000	NC	NA		NA	21	NC	NC	NC

[a]<LOD: below limit of detection [b]NA: not analyzed [c]NC: not calculated
[d]The average of the three low F substrates (22400) was used for calculations since equal masses of each were added to the duplicate low F reactors
The summed PFAS includes suspect compounds.

Table S13. Summed PFAS as F released in leachate and ratio of leachate F to gas phase F.

Reactor group	Summed F of ionic PFAS released in leachate (ng)	Total F of ionic PFAS released in leachate/total F released in gas (%)
high F-Natural Plates (a,b,c)	21800	40
	34400	101
	27700	51
high F-Compostable Bowls	15500	78
high F-Biodegradable Boxes	17100	53
high F-Bagasse Containers	16100	195
high F-Eco-friendly Plates	1677	51
high F-Popcorn Bag 2	102	3
Low F-mixed paper plates, eco-friendly food trays, polycoated freezer paper	64	2145
	31	496
MSW-May (a, b)	928	5963
	20	116
MSW-August (a, b)	95	314
	44	414

Figure S1. Schematic of the test reactor. **A.** 2L-glass reactor with flange connection part; **B.** drainage layer of pea gravel; **C.** #120 stainless steel woven wire mesh to reduce clogging of the reactor outlet; **D.** glass cap for the reactor with flange connection part; **E.** flange clamps to hold the two-pieces of glass together and keep the system sealed; a 245 EPDM gasket was placed between the two pieces of glass; **F.** stainless steel adjustable clamp; **G.** 10 mL Norm-Ject Luer lock polypropylene syringe; **H.** T-shape plastic 3-way barb fitting, polypropylene, 1/4" OD; **I.** Cole-Parmer female/male Luer hose barb adapter, 1/4" OD; **J.** McMaster quick-disconnect tube couplings, polysulfone, 1/4" OD; **K.** tubing flow control hose clamp, 3/4" OD 54; **L.** stainless steel quick-turn tube couplings, 1/4" OD; **M.** one-way Luer stopcock; **N.** tygon tube, 1/4" ID, flexible, clear, PVC; **O.** 10 L-foil gas bag (SKC. Inc.), **P.** 2L-Thermo Scientific™ Labtainer™ bioProcess container, with 3 Ports, MPC Insert, MPC Body, and Luer lock.

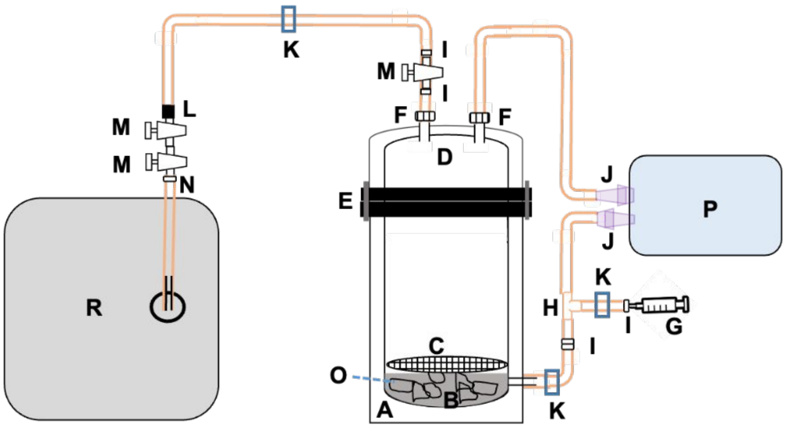

Figure S2. Gas sampling system. **A**. 0.5 L-foil gas bag; **B.** check valve; **C.** 160 mL-serum bottle; **D.** H_2SO_4 aqueous solution (pH <3.7), 1 g/L methyl orange was added as a pH indicator (3.1-4.4); **E.** 18-gauge needles; **F.** rubber stopper to seal serum bottle. **G.** straight adapters, tube push-on barbed hose, 1/4" ID; **H**. Markes Universal gas sampling tube 1/4" OD (IS spiked TD tube); **I**. One-way Luer stopcock.

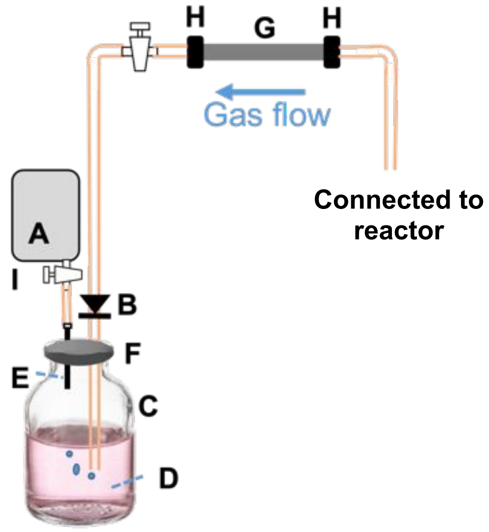

Figure S3. Measured PFAS concentrations in the Whatman #2 filter paper Control reactors. **A**. Control 1 reactor and **B**. Control 2 reactor.

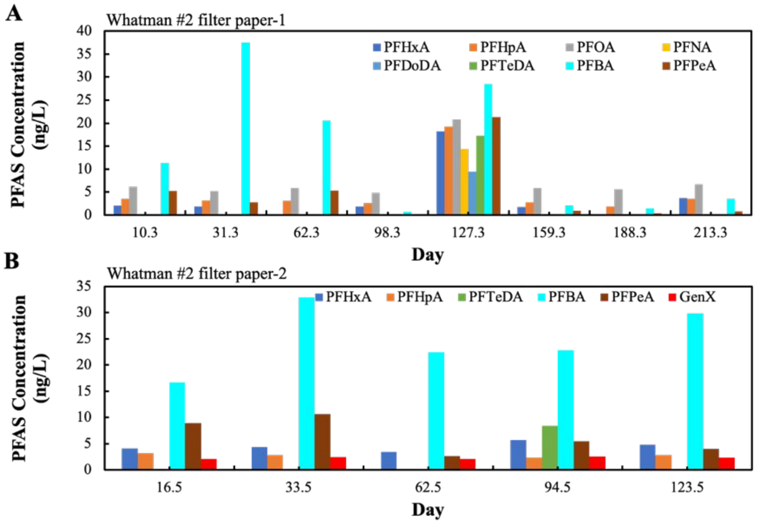

Figure S4. pH of all reactors: **A.** Control and Low F; **B.** MSW; **C.** High F (Natural Plates and Popcorn Bags), and **D.** High F (Compostable Bowls, Biodegradable Boxes, Bagasse Containers, and Eco-friendly Plates).

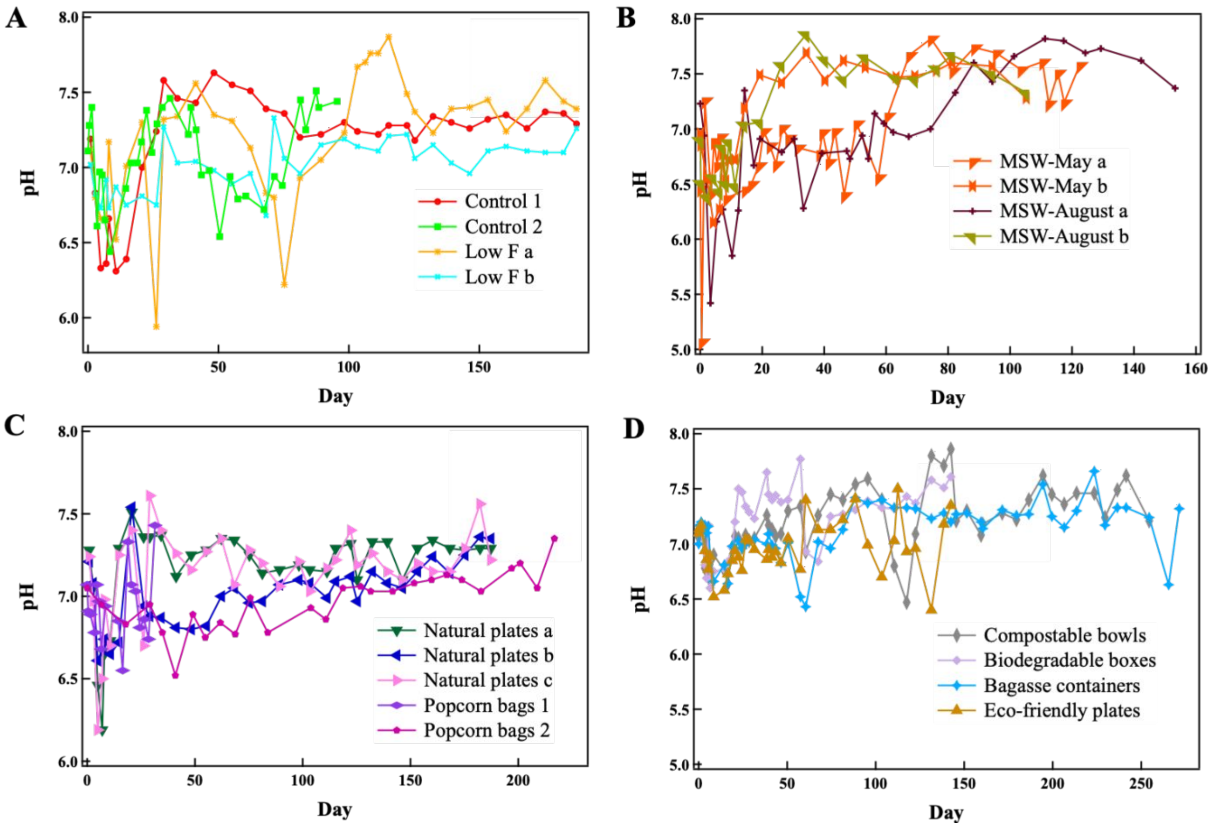

Figure S5. CH_4 yield in the reactors of **A.** Whatman #2 filter paper control; **B.** MSW; **C.** Popcorn bags, and **D.** High F (Compostable Bowls, Biodegradable Boxes, Bagasse Containers, and Eco-friendly Plates).

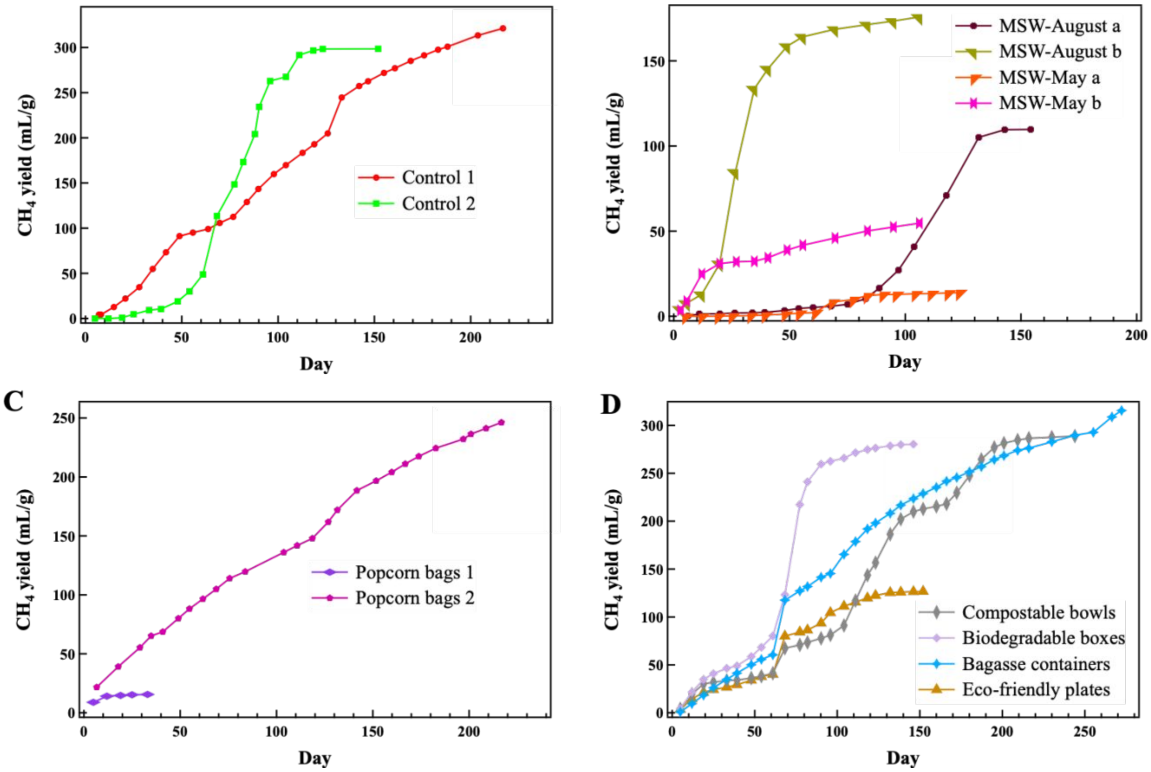

Figure S6. CH_4 generation rate (mL/day-dry g) of all reactors: **A.** Control and Low F; **B.** MSW; **C.** High F (Natural Plates and Popcorn Bags), and **D.** High F (Compostable Bowls, Biodegradable Boxes, Bagasse Containers, and Eco-friendly Plates).

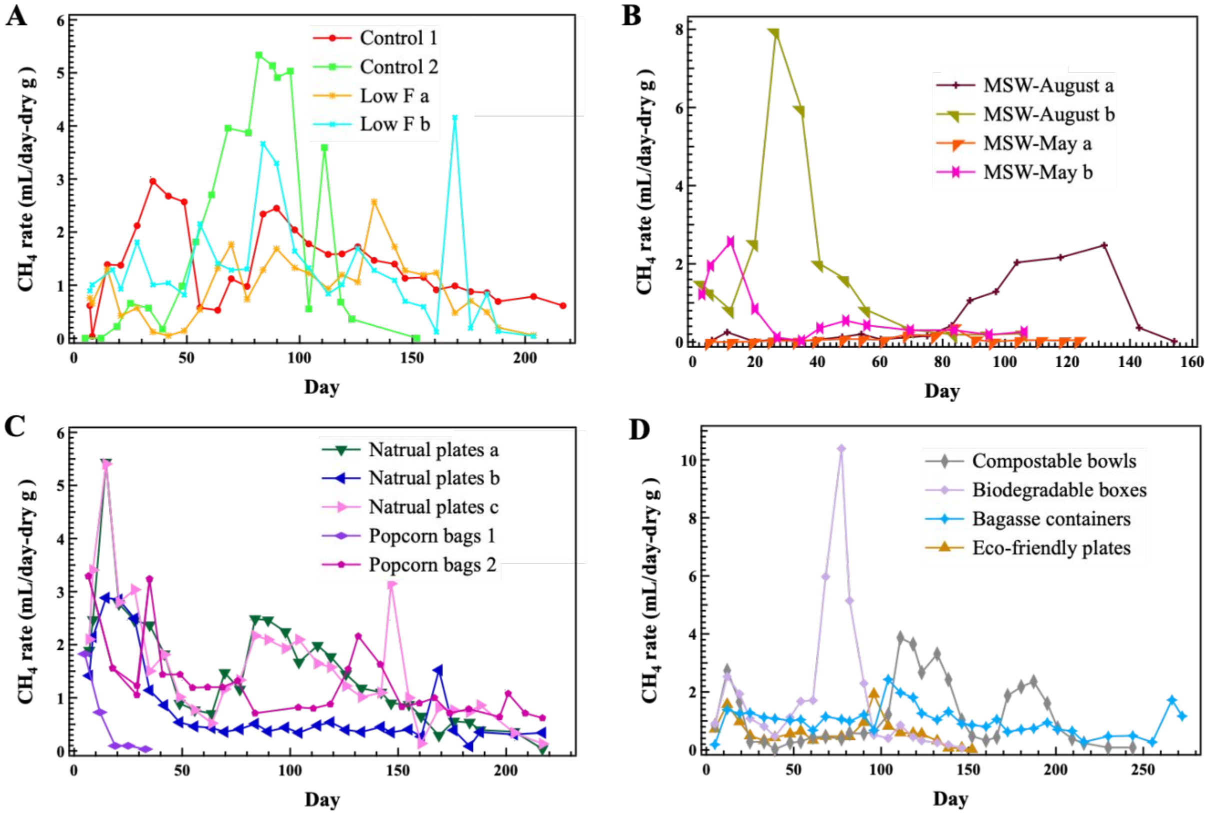

Figure S7. The total fluorine concentration of 46 tested materials (5 popcorn bags sampled in 2022 were not tested by PIGE).

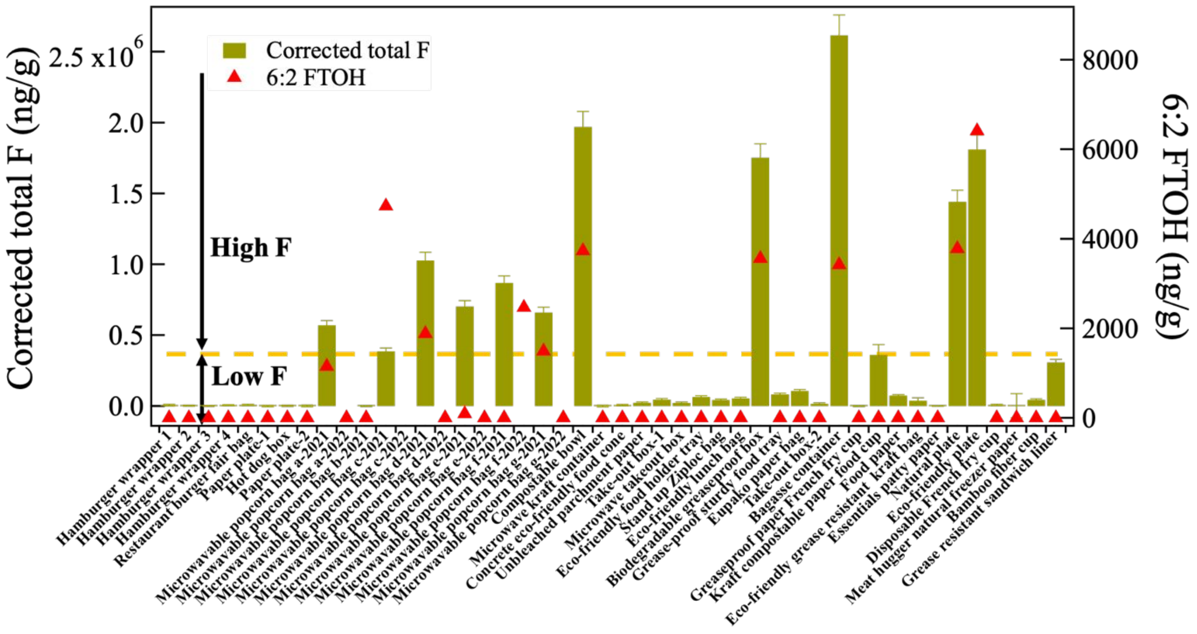

Figure S8. Volatile PFAS released from Popcorn Bags 1 reactor during the 27-day monitoring period. PFAS data are presented as a yield. As such, a flat line indicates that no PFAS was detected in a sample. The first gas sampling and detection day was day 12, and concentrations before day 12 are extrapolated.

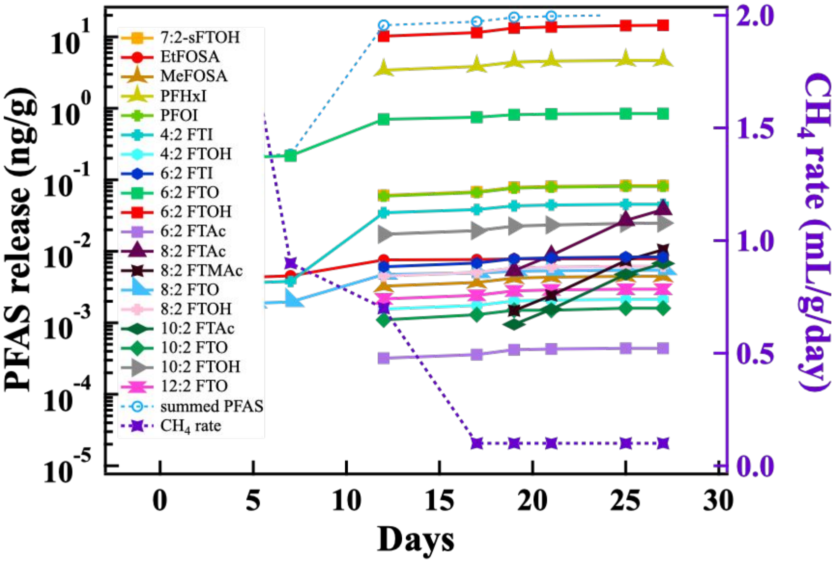

Figure S9. Time-dependent release of volatile PFAS to the gas phase in **A.** Natural Plates a and **B.** Natural Plates b. The summed PFAS and 6:2 FTOH lines overlap in both reactors.

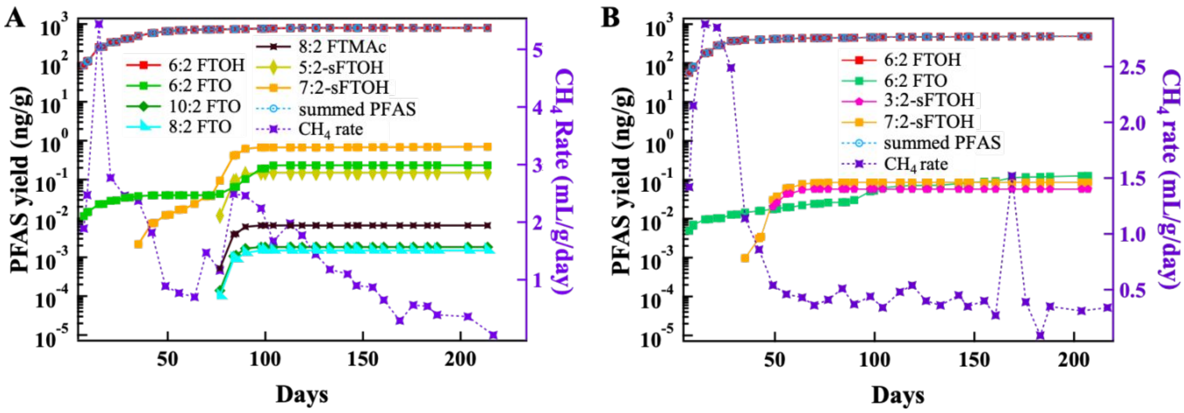

Figure S10. Volatile PFAS released from High F reactors. **A**. Compostable Bowls; **B.** Biodegradable Boxes; **C.** Bagasse Containers; **D.** Eco-friendly plates. The summed PFAS and 6:2 FTOH lines overlap in all reactors.

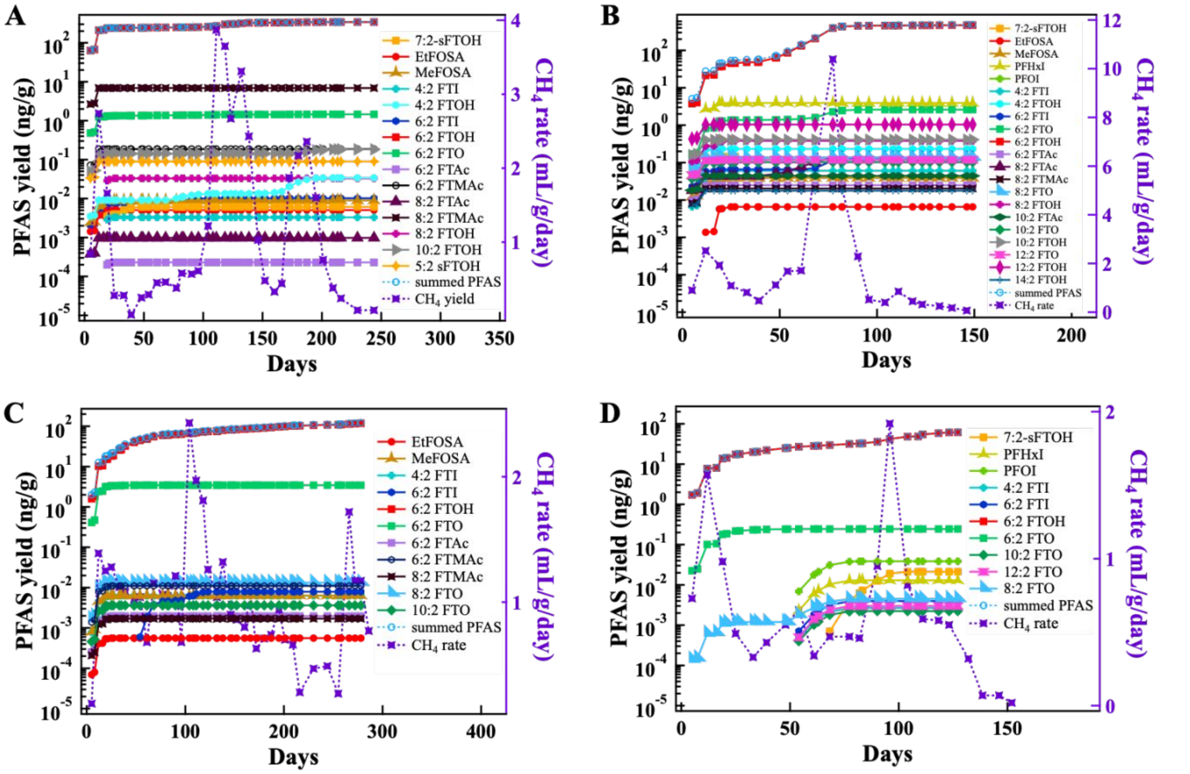

Figure S11. Time-dependent release of volatile PFAS in **A**. Low F a, **B**. Low F b, **C**. MSW-August b, and **D**. MSW-May a reactor. The concentration of 7:2-sFTOH was first detected on day 74 for Low F a reactor and day 51 for Low F b reactor, 8:2 FTOH were first detected on day 43 in Low F b reactor, 10:2 FTOH were first detected on day 148 and 43 in Low F a and Low F b reactors; yields of these compounds before first detection were extrapolated. The summed PFAS and 7:2-sFTOH lines overlap in Figure S11C.

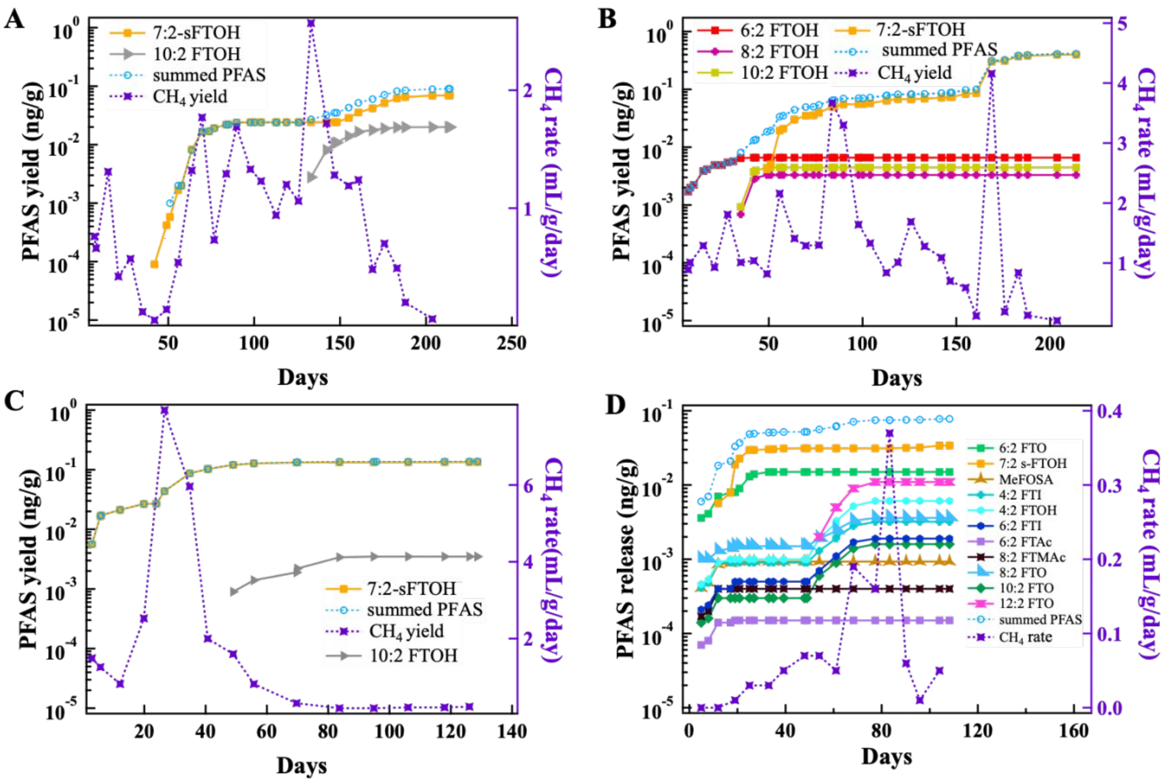

Figure S12. PFAS concentration and relative abundance in leachate samples over time for the High F reactors: **A&B**. Compostable Bowls, **C&D**. Biodegradable Boxes, **E&F**. Bagasse Containers, **G&H**. Eco-friendly Plates, **I&J**. Popcorn Bags 1, and **K&L**. Popcorn Bags 2.

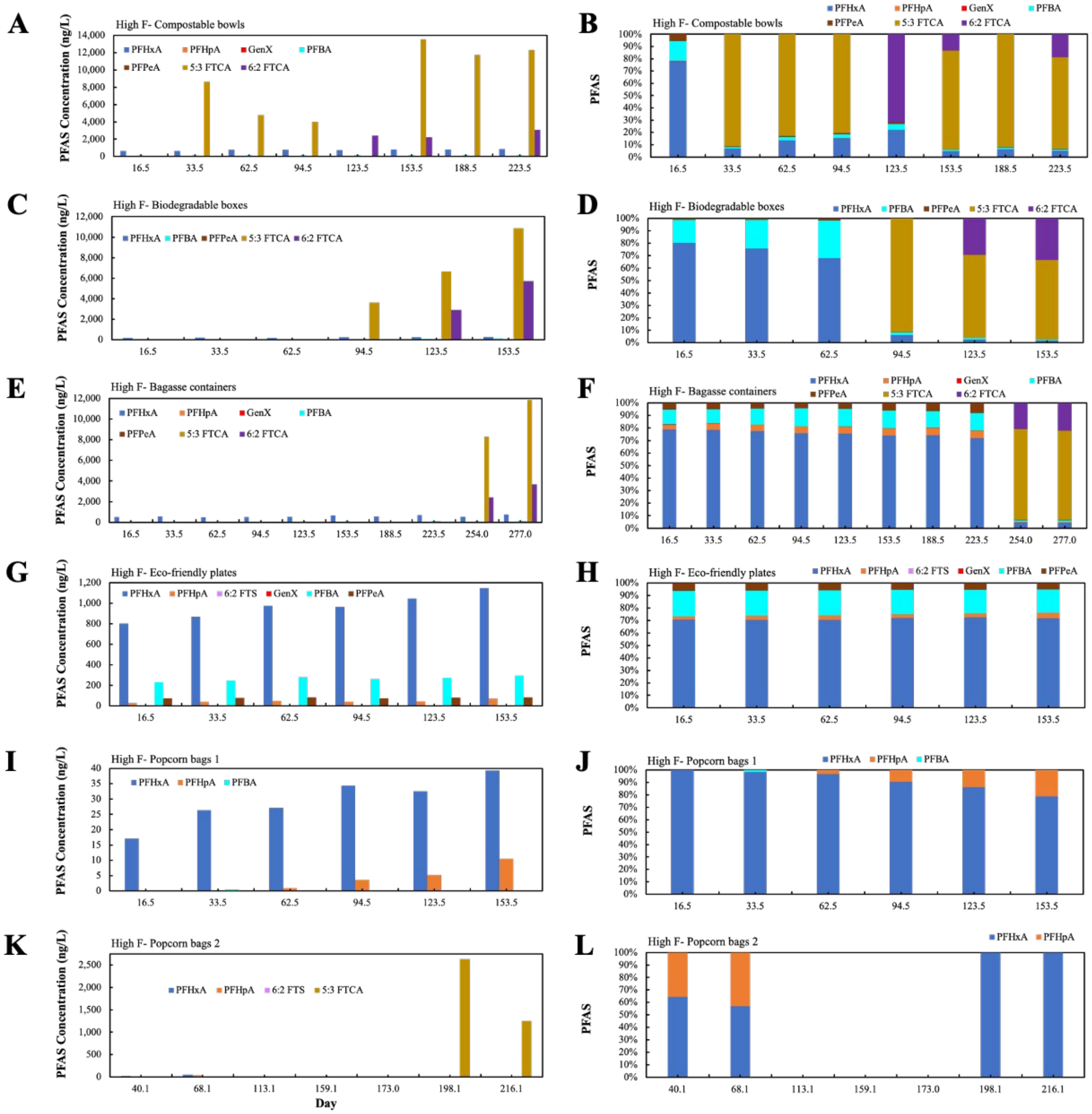

Figure S13. PFAS concentration and relative abundance and CH_4 generation rate in the leachate samples over time for: **A&B**. High F Natural Plates a, **C&D**. High F Natural Plates b, and **E&F**. High F Natural Plates c. The discontinuous reporting of FTCAs concentrations in Natural Plates a could be due to the high LOQ of 5:3 FTCA (2500 ng/L) and 6:2 FTCA (2500 ng/L).

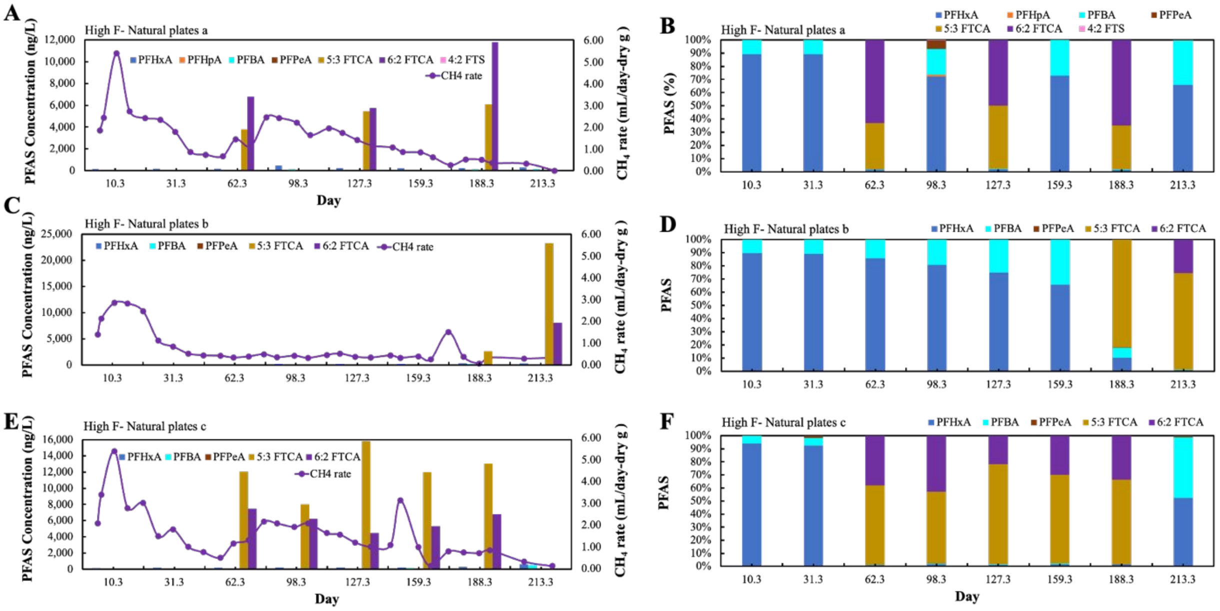

Figure S14. The PFAS concentration and relative abundance of each PFAS component in the leachate samples of **A&B.** Low F a **C&D.** Low F b.

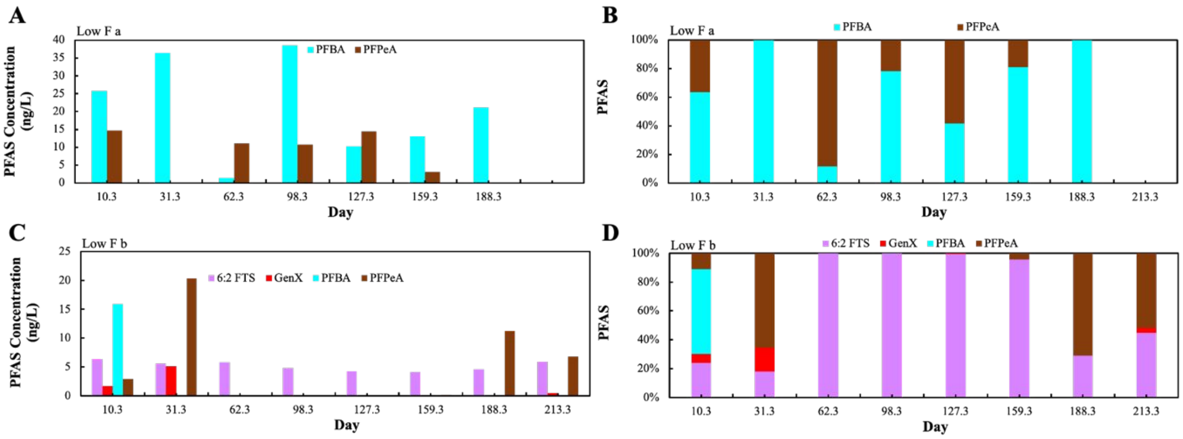

Figure S15. The PFAS concentration in the leachate samples of **A.** MSW-May a, **B.** MSW-May b, **C.** MSW-August a, and **D.** MSW-August b. In the case of PFOA, concentrations were 22 to 57% above the highest concentration in a control reactor.

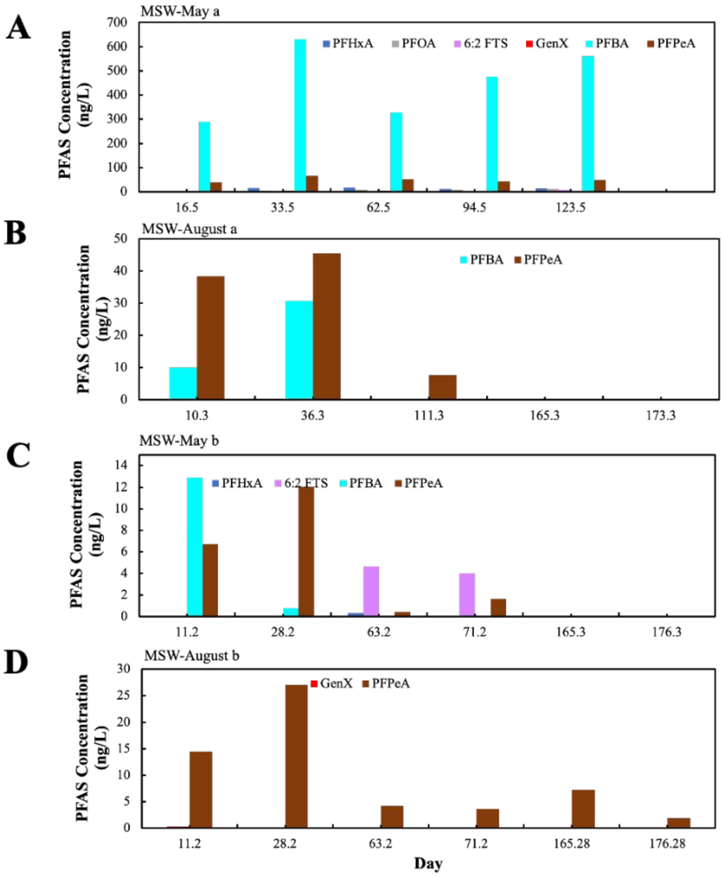

Figure S16 A. Cumulative summed and individual ionic PFAS released to leachate from each reactor. **B.** The fraction of each PFAS released to the leachate.

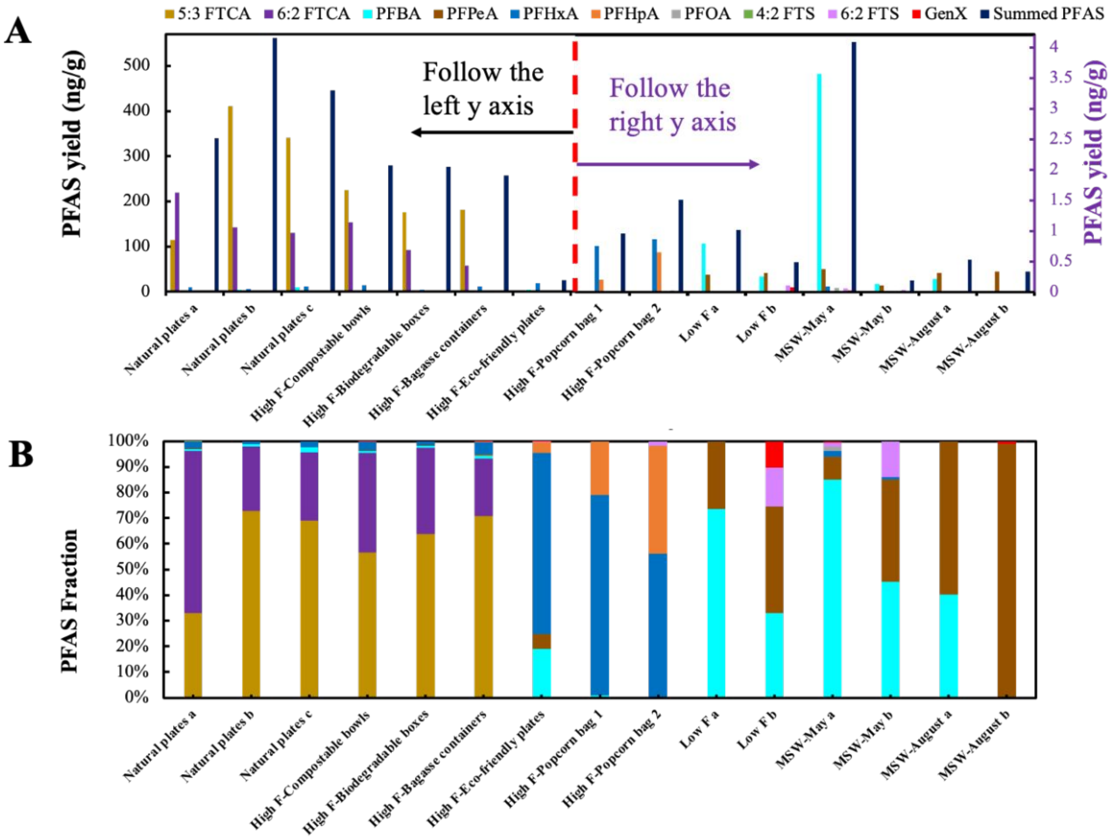

Supplement: Supplementary file 2 — es4c08544_si_002.pdf [file es4c08544_si_002.pdf]
